# Increasing Cervical Cancer Screening Coverage: A Randomised, Community-Based Clinical Trial

**DOI:** 10.1371/journal.pone.0170371

**Published:** 2017-01-24

**Authors:** Amelia Acera, Josep Maria Manresa, Diego Rodriguez, Ana Rodriguez, Josep Maria Bonet, Marta Trapero-Bertran, Pablo Hidalgo, Norman Sànchez, Silvia de Sanjosé

**Affiliations:** 1 Atenció a la Salut Sexual i Reproductiva (ASSIR) SAP Cerdanyola–Ripollet, Institut Català de la Salut, Ripollet, Spain; 2 Unitat de Suport a la Recerca Metropolitana Nord. Institut de Investigació en Atenció Primària (IDIAP) Jordi Gol. Sabadell, Spain; 3 Departament de Medicina, Universitat de Barcelona. Barcelona, Spain; 4 Grup de Recerca en Atenció Sexual i Reproductiva IDIAP Jordi Gol. Sabadell, Spain; 5 Departament de Infermeria. Universitat Autònoma de Barcelona. Bellaterra, Cerdanyola, Spain; 6 Servei d’Atenció Primària SAP Vallés Occidental. Institut Català de la Salut, Sabadell, Spain; 7 Center for Research in Economics and Health (CRES). University Pompeu Fabra, Barcelona, Spain; 8 Universidad de Castilla La Mancha. Ciudad Real, Spain; 9 Cancer Epidemiology Research Programme | IDIBELL | Catalan Institute of Oncology, L’Hospitalet de Llobregat, Spain; 10 Centro de Investigación Biomédica en Red en Epidemiologia y Salud Pública CIBERESP, Barcelona, Spain; Universita degli Studi di Roma Tor Vergata, ITALY

## Abstract

**Background:**

Opportunistic cervical cancer screening can lead to suboptimal screening coverage. Coverage could be increased after a personalised invitation to the target population. We present a community randomized intervention study with three strategies aiming to increase screening coverage.

**Methods:**

The CRICERVA study is a community-based clinical trial to improve coverage of population-based screening in the Cerdanyola SAP area in Barcelona.A total of 32,858 women residing in the study area, aged 30 to 70 years were evaluated. A total of 15,965 women were identified as having no registration of a cervical cytology in the last 3.5 years within the Public Health data base system. Eligible women were assigned to one of four community randomized intervention groups (IGs): (1) (IG1 N = 4197) personalised invitation letter, (2) (IG2 N = 3601) personalised invitation letter + informative leaflet, (3) (IG3 N = 6088) personalised invitation letter + informative leaflet + personalised phone call and (4) (Control N = 2079) based on spontaneous demand of cervical cancer screening as officially recommended. To evaluate screening coverage, we used heterogeneity tests to compare impact of the interventions and mixed logistic regression models to assess the age effect. We refer a “rescue” visit as the screening visit resulting from the study invitation.

**Results:**

Among the 13,886 women in the IGs, 2,862 were evaluated as having an adequate screening history after the initial contact; 4,263 were lost to follow-up and 5,341 were identified as having insufficient screening and thus being eligible for a rescue visit. All intervention strategies significantly increased participation to screening compared to the control group. Coverage after the intervention reached 84.1% while the control group reached 64.8%. The final impact of our study was an increase of 20% in the three IGs and of 9% in the control group (p<0.001). Within the intervention arms, age was an important determinant of rescue visits showing a statistical interaction with the coverage attained in the IGs. Within the intervention groups, final screening coverage was significantly higher in IG3 (84.4%) (p<0.001). However, the differences were more substantial in the age groups 50–59 and those 60+. The highest impact of the IG3 intervention was observed among women 60+ y.o with 32.0% of them being rescued for screening. The lowest impact of the interventions was in younger women.

**Conclusions:**

The study confirms that using individual contact methods and assigning a fixed screening date notably increases participation in screening. The response to the invitation is strongly dependent on age.

**Trial Registration:**

ClinicalTrials.gov NCT01373723

## Introduction

Despite numerous recommendations calling for the implementation of organised cancer screening programmes, cervical cancer screening remains opportunistic in many European countries. In Spain, the age-standardized incidence rate of cervical cancer remains relatively low compared to many European countries with an estimated rate around 7.8 per 10^5^ women for 2012 and a mortality rate of 2.1 per 10^5^[1–3]. However, cervical cancer mortality rates have stopped to decline in the last decade [4].

Screening is largely opportunistic and free of charge within the Spanish Public Health system. In the autonomous region of Catalonia, several actions have been implemented since 2006 to increase coverage of select populations [5]. Analysis of the Catalonian data has revealed poor regular attendance, of once screened women, and high prevalence of cervical lesions in women high poor screening history [6–7]. Some studies from Spain have reported that between 50–80% of women with invasive cervical cancer had not undergone cytology testing during the 10 years prior to their cancer diagnosis [8–10]. Consequently, an improved screening coverage seems to be necessary to observe a reduction in the mortality attributable to cervical cancer. This is in line with European guidelines and other scientific societies including Spanish and Catalan ones, recommend an organized approach of ‘call and recall’ of the target population[1,11–14]. However, a recent report analyzing the situation of cervical cancer screening in Europe identified a large number of countries lacking a standardized approach for quality assurance, for monitoring or evaluation of the country based strategies to prevent cervical cancer [2]. These data suggest the need to further advocate for organized screening and to investigate different approaches to facilitate the transition from opportunistic to organized screening.

In this report, we aimed to evaluate the overall impact of personalized invitations to attend cervical cancer screening.

## Methods

The CRICERVA study [15, 16] is a cluster randomised community-based clinical trial implemented within the Cerdanyola Primary Health Care Service (SAP Cerdanyola). SAP Cerdanyola is a predefined geographical area located in the metropolitan belt of Barcelona, Spain (**S1 File** Published protocol **S2 File**.- Study protocol Spanish, **S3 File**.- Study protocol English translation, **S4 File**.- Consort Checklist). It is subdivided into five Basic Health Care Centers, four of which were included in the CRICERVA study. The fifth one is an upper- class residential area, socially very distinctive from the other areas, and excluded in this study.

At the time of the study, SAP Cerdanyola covered a population of 120,293 individuals, of which 72,036 were women and 32,858 were within the screening target ages 30–70. As part of the National Health Service (NHS), residents in a given area have assigned a general practitioner (GP) and a set of specialists including gynecologists. Above 95% of the population is covered by the NHS and, within a 5 year interval, above 90% of the women have had a contact with the assigned GP [6].

**Fig 1:**
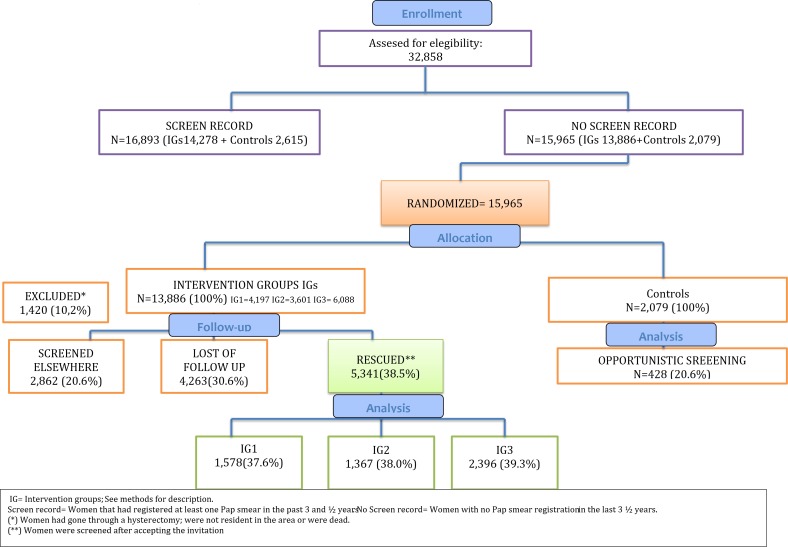
Study flow diagram.

For the study purposes, eligibility included women whose GP was assigned to the Cerdanyola SAP area, were resident in the area for more than 6 months and had no record of a cervical cytology result in the medical registry of cervical cancer screening in the last 3.5 years” (Fig 1). Medical records were reviewed searching for the date of the last available cervical cytology result and 15,965 women with no cervical cytology records were identified. At this point, the four Basic Health Care Centers were cluster-randomized into the different intervention groups: including: The Intervention Group 1 (IG1) including 4197 women that received a personalised invitation letter, the Intervention Group 2 (IG2) including 3601 women that received a personalised invitation letter plus and informative leaflet, the Intervention Group 3 (IG3) including 6088 women that received a personalised invitation letter plus the informative leaflet and a personalised phone call three days before the appointment and finally a Control group including 2079 women in which no action was taken and whose screening was based on spontaneous demand during the study period. Women in the IGs were invited to participate accordingly to the study arm. Generally women contacted the Basic Health Care Centers by phone to confirm or to cancel the proposed appointment. At the first verbal contact with the women, the adequacy of the screening history was evaluated and if women referred to have a private gynecologist for cervical cancer screening visits, the date of her last visit was noted and the appointment was cancelled. Further, women were excluded for a screening visit if they were not residents in the area, had undergone a hysterectomy, had a current history of cervical disease; were HIV positive, immunosuppressed or dead. A total of 1420 were excluded. About 2% of the letters were returned for erroneous address. Women selected for screening were verbally informed about the screening procedures and the significance of the results. Women lost to follow up were phoned at least 3 times after the field work of the study had finished.

No changes in eligibility criteria were observed throughout the study.

Opportunistic cervical cancer screening visits in the control group were registered during the study period for comparison with the IGs.

The record search of the patients was initiated in January 2011, women were contacted when the ethical approval was granted (May 2011) and the study trial was registered (June 2011). Recruitment started in June 2011 and finished in December 2013.

## Statistical Analysis

Population-based coverage was estimated at baseline and after the intervention. Baseline coverage relates to a 3.5 year period. Final coverage computes the baseline screening estimated through our records plus the screening referred to take place within the private sector plus the screening obtained through the rescue visit. Coverage estimates were stratified by age and by intervention group and. Overall coverage was calculated by age-weighted for each arm. Chi-square tests were used to compare intervention and control groups in terms of participation.

Mixed logistic regression models were used to estimated how age impacted in the rescue proportion with the intervention group as a random effect and the age as categorical fixed effect This analysis was restricted to participants in the three intervention groups and excluding women who were screened in private system,. Interaction between age and intervention group was assessed by comparing the model with and without the interaction term using the log-likelihood ratio test.

The level of significance used was p ≤0.05. Analyses were performed using SPSS for Windows v. 20.0. and R (version 3.2.5) with the lme4 package [17].

## Ethics Approval

Approval of this study was granted by the Ethics Committee of the IDIAP Jordi Gol. No written signature was requested to participants as the study involved regular clinical practice. This was approved by the Ethical Committee.

After the study was completed, we proceeded to call women in the control group that had not requested a screening visit during the study period and that were eligible for screening.

Through the text we refer to ‘rescued’ women those that had insufficient screening uptake and that through our personalized contact accepted to undergo screening for cervical cancer.

## Results

Table 1 summarizes the different components of coverage in the 26,744 women targeted in the intervention groups(IGs) and 4,694 women in the control group (Table 1). All women in the IGs received an invitation letter with a specific date of the visit.

**Table 1:** Cervical cancer screening coverage at baseline, those referring a private gynecologist and rescued coverage post intervention and final population coverage attained by intervention groups and the control group at five and a half years*.

	IG1		IG2		IG3		TOTAL IGs		CONTROL	
	N	%	N	%	N	%	N	%	N	%
**TARGET POPULATION**	8504	100	7842	100	10398	100	26744	100	4694	100
BASELINE COVERAGE	4771	56.1	4568	58.3	4939	47.5	14278	53.4	2615	55.7
**STUDY TARGET POPULATION**	4197	49.3	3601	45.9	6088	58.5	13886	51.9	2079	44.3
PRIVATE SCREENING	778	9.1	649	8.2	1435	13.8	2862	10.7	NK	
RESCUED^&^	1578	18.6	1367	17.4	2396	23.0	5341	20.0	428	9.1
**Increased coverage**^$^	27.8%		26.2%		37.3%		31.0%		9%	
**FINAL COVERAGE**	7127	83.8	6584	84.0	8770	84.4	22481	84.1	3043	64.8
*LOST TO FOLLOW UP*	*1377*	*16*.*1*	*1258*	*16*.*0*	*1628*	*15*.*7*	*4263*	*15*.*9*	*NK*	
P value heterogeneity test in final coverage & lost to follow up	**P = 0.5807**					NA				
P value heterogeneity test in rescue & target population	**P = 0.000**					**P = 0.000**				

NK = Unknown NA = Not applicable. All percentages are referred to the Target Population.

& = For control group attendance to screening was opportunistic.

$ = Weighted coverage by age structure of IGs.

*The table excludes *1420 women because of hysterectomy for benign reasons*, *changes of residence and death*.

Amon the IGs, 5,341(20.0%) women had the study criteria to undertake a rescue screening visit. There was no difference in the percent recruitment of this group across IGs. Further, 2,862 (10.7%) women call us back or come to the health center referring to have had a regular cytology within the private sector. These women were registered as having an adequate screening and no further action was taken with them. This was more commonly reported by target women in IG3 (13.8%) than by those in IG1(9.1%) or IG2 (8.2%) (p value <0.001). Finally, 4,263 (15.9) women were lost to follow-up with no differences across the IGs (p = 0.58). Within the control group, opportunistic screening was registered in 428 (9.1%) women during the study period.

All intervention strategies significantly increased participation to screening compared to the control group. Coverage after the intervention reached 84.1%, while coverage in the control group reached 64.8% (Table 1). The final impact of our study resulted in an increase of 20% from the rescue components in the three IGs and of 9% in the control group (p<0.001). Within IGs, final screening coverage was significantly higher in IG3 (84.4%) (p<0.001) which represented an overall age-adjusted increase of our coverage estimation of 37.3%.

The analysis was consistent in the crude approach and using the log-likelihood ratio test comparing the null model (no variables) with the model including the intervention group (p<0.001).

Within IGs, age was an important determinant of rescue visits showing a statistical interaction with attained coverage. The comparison between the IGs and age in four categories model showed to be highly significant, as well as it was the interaction between age and IGs (p<0.001). Thus, coverage in the IGs could be better interpreted by age strata as described in Table 2. Although IG3 reached higher rescue estimates in all intervention groups, the differences were more substantial in the age groups 50–59 and those 60+. The highest impact of IG3 was observed among women 60+ y.o with 32.0% of the target population being rescued for screening. The lowest impact of the interventions were observed in younger women with no difference by IGs.

**Table 2:** Cervical cancer screening coverage at baseline, those referring a private gynecologist and rescued coverage post intervention and final population coverage attained by intervention and age groups.

									Age group															
			<40						40–49						50–59						≥60			
	IG1		IG2		IG3		IG1		IG2		IG3		IG1		IG2		IG3		IG1		IG2		IG3	
	N	%	N	%	N	%	N	%	N	%	N	%	N	%	N	%	N	%	N	%	N	%	N	%
**TARGET POPULATION**	3156	100	2775	100	3643	100	2376	100	2107	100	2705	100	1677	100	1824	100	2263	100	1295	100	1136	100	1787	100
BASELINE COVERAGE	1862	59.0	1647	59.4	1939	53.2	1343	56.5	1260	59.8	1204	44.5	977	58.3	1131	62.0	1158	51.2	589	45.5	530	46.7	638	35.7
**STUDY TARGET**	1294	41.0	1128	40.6	1704	46.8	1033	43.5	847	40.2	1501	55.5	700	41.7	693	38.0	1105	48.8	706	54.5	606	53.3	1149	64.3
RESCUED	449	14.2	392	14.1	576	15.8	512	21.5	381	18.1	665	24.6	314	18.7	318	17.4	584	25.8	303	23.4	276	24.3	571	32.0
PRIVATE SCREENING	415	13.1	266	9.6	625	17.2	172	7.2	164	7.78	451	16.7	89	5.3	104	5.7	173	7.6	102	7.9	115	10.1	186	10.4
LOST TO FOLLOW UP	430	41.0	470	16.9	503	13.8	349	14.7	302	14.3	385	14.2	297	17.7	271	14.9	348	15.4	301	23.2	215	18.9	392	21.9
**FINAL COVERAGE**	3002	95.1	2557	92.1	3551	97.5	1904	80.1	1717	81.5	2178	80.5	1389	82.8	1568	86.0	1903	84.1	832	64.2	742	65.3	1138	63.7
p-value heterogeneity IG1-IG2-IG3			0.554						0.029						0.001						0.001			

## Discussion

To our knowledge, this is the first population-based randomized trial designed to increase cervical cancer screening coverage in Catalonia. The study confirmed that an active search for women to undergo screening for cervical cancer considerably increased participation rates among all age groups and particularly so among women over age 49. Coverage post-intervention was 34% higher than that observed under strict opportunistic screening. This improvement was likely due to the provision of better information regarding screening and the invitation to participate and this had a relevant effect among the elder target women. No major absolute differences were observed between interventions strategies used. The trial identified a substantial number of women that had never had a screening test, adding additional value to the study intervention and confirmed a proportion of women attending private practice.

A letter with a fixed appointment for the three study arms was set up across the intervention arms following previous recommendations [13,14]. In addition, (a) information regarding cervical cancer and cervical cancer screening and (b) a personalized telephone call were included in the different arms. The coverage reached in these groups was compared to that of the control group in which the official recommendations were implemented. That is, women in the control group were not called for a screening test. In all circumstances a negative cytology was followed by a 3 year interval for the next cytology test.

In the IGs there was a substantial overall increase in cervical cytology coverage during the study period. Coverage went from 53.4% at baseline to 84.1% at the end of the study. The increase in coverage was of a similar magnitude in the three intervention study arms, although the percentage increase was higher for IG3 with a slightly higher final coverage of 84.4% compared to the overall estimate. These results are consistent with a previous study in Spain in which interventions based on written or telephone briefing were effective to increase a two year cytological coverage compared to the control group [15].

When we evaluated the changes in coverage by age groups we observed that women in the age groups 50–59 and 60+ showed the largest net benefit with increased coverage values of 21.2% and 27.3% respectively. Contrary, the baseline coverage for women younger than 40 was slightly higher and had a higher reporting of screening in the private sector compared to other age groups. This resulted in a statistically significant lower impact of the study invitation among this age group (14.8%) compared to the remaining age groups in which the coverage increased from 70.5% to 85.3% (p<0.001). This baseline higher screening coverage among the youngest is likely to be explained by the higher contact with reproductive health care centers and implying that women are more likely to be offered a screening test together with other medical services related to contraception or reproduction. Although our control arm showed poorer screening uptake compared to the IGs (84.1%) the final coverage was higher (64.8%) than the average reported for Catalonia [16]. This can be explained by the fact that the four areas included in the study from SAP Cerdanyola have traditionally shown to have a good connection with the NHS. These areas are represent a metropolitan urban environment with high predominance of working class, where half of the women have a primary school completed or less and show an almost universal use of the Public Health Services compared to other areas in Catalonia [16].

The answer to the invitation letter overall was 59.1% which compared to other efforts to contact non-attenders can be considered high [18]. Our data is in line with a study in Italy in which 23–58% of the women first invited to participate in the screening, accept, while second remainder for those lost-to follow up dropped substantially the acceptance with a response rate for a cervical pap was 11% and was lower in the elderly population [19]. As we have noted in the analysis of women aged 60 and older [15], the fact that the letters were signed by the family physician and the coordinators of the BHCC, who are generally professionals known by the target population added to the overall coordination of the activity and probably helped to increase attendance as observed by others [18]. However, when women lost to follow-up were contacted by phone at the end of the study only 22% could be contacted (Manuscript in preparation). Multiple strategies are now under evaluation to reach these hard to reach women including self-sampling approaches that may increase substantially the rescue of an additional proportion of women [19–23].

Quality of the available information is reported to be key in influencing better allocation of resources and better participation in screening [2, 14,24]. Part of this work could be done because of the availability of eCAP, which provides individual medical histories in an electronic format and gives information on all activities occurring at the primary health care level and it can be linked to cytology records. eCAP is also continuously updated. However, eCAP does not always include hospital data, death records or assistance outside the public health care system. Thus, the fact that we interviewed all the women participating in the study allowed us to remove those women that did not need or were not eligible for screening. We are now exploring the possibility of improving some of the data query in eCAP so that it can be used as a tool in the forthcoming recommendations for cervical cancer screening.

The study is subject to several limitations. The study aimed to reach all women at risk of cervical cancer in the study population. However, 16% of the women could not be contacted as they never responded to our letter or telephone call. We do not know the exact reasons for this as only 2% of the letters were returned to us due to a faulty addresses Although we had a good response to the first phone contact in the intervention group including a telephone call, IG 3, some women reacted negatively against being contacted by phone due to the huge number of calls that they receive, often from sales people. This should be considered if a telephone contact is recommended in future strategies. Maybe there is a group of women that cannot understand the purpose of our invitation or that they do not want to be told what to do in terms of health issues. Further work is needed in this topic.

Further, women with no screening records were asked about regular screening outside the NHS. We could not verify the records of those women referring attending regular screening in a private practice.

The study is by design a cluster randomized trial that would require a full multilevel model analysis. This could only be done within IGs controlling by the influence of age. For the whole population we had only aggregated information.

Taking into account the limitations mentioned above, the study supports the recommendation that screening coverage can substantially be increased under an organized approached to personally contact subjects at risk. Finally, in a concomitant cost-effectiveness analysis of the three trial interventions IG1, IG2 and IG3, the most cost-efficient intervention to increase the uptake of cervical screening across ages compared with the opportunistic approach was sending an invitation letter (IG1), specially to women ≥60 years. IG2 was more costly for the benefits in coverage and IG·3 although was the best in coverage was also the one that used more economical resources [25] (S5 File.- Submitted related work).

## Conclusions

The study confirms that using individual contact methods and assigning a fixed screening date notably increases participation in screening. The response to our invitation process was strongly dependent on age. The findings from this study could be used to facilitate the implementation of effective population-based strategies toward the transition from opportunistic to organised cervical cancer screening.

## Supporting Information

S1 File Published Protocol. (PDF)

S2 File Study protocol Spanish. (PDF)

S3 File Study protocol English translation. (DOC)

S4 File Consort Check list. (DOC)

S5 File Submitted related work. (PDF)
