# Supplementary material for: Increasing Cervical Cancer Screening Coverage: A Randomised, Community-Based Clinical Trial
Source: PLoS One. 2017 Jan 24;12(1):e0170371. doi: 10.1371/journal.pone.0170371 (PMC5261609; doi:10.1371/journal.pone.0170371)
Supplement: S1 File — (PDF) [file pone.0170371.s001.pdf]

Acera et al. BMC Health Services Research 2011, 11:278
http://www.biomedcentral.com/1472-6963/11/278

BMC Health Services Research

STUDY PROTOCOL

Open Access

Economic evaluation of three populational screening strategies for cervical cancer in the county of Valles Occidental: CRICERVA clinical trial

Amelia Acera[1,4*], Ana Rodriguez[1], Marta Trapero-Bertran[2], Pilar Soteras[1], Norman Sanchez[5], Josep M Bonet[3], Josep M Manresa[4,7], Pablo Hidalgo[3], Pere Toran[4] and Gemma Prieto[6]

Abstract

Background: A high percentage of cervical cancer cases have not undergone cytological tests within 10 years prior to diagnosis. Different population interventions could improve coverage in the public system, although costs will also increase. The aim of this study was to compare the effectiveness and the costs of three types of population interventions to increase the number of female participants in the screening programmes for cancer of the cervix carried out by Primary Care in four basic health care areas.

Methods/Design: A cost-effectiveness analysis will be performed from the perspective of public health system including women from 30 to 70 years of age (n = 20,994) with incorrect screening criteria from four basic health care areas in the Valles Occidental, Barcelona, Spain. The patients will be randomly distributed into the control group and the three intervention groups (IG1: invitation letter to participate in the screening; IG2: invitation letter and informative leaflet; IG3: invitation letter, informative leaflet and a phone call reminder) and followed for three years. Clinical effectiveness will be measured by the number of HPV, epithelial lesions and cancer of cervix cases detected. The number of deaths avoided will be secondary measures of effectiveness. The temporal horizon of the analysis will be the life expectancy of the female population in the study. Costs and effectiveness will be discounted at 3%. In addition, univariate and multivariate sensitivity analysis will be carried out.

Discussion: IG3 is expected to be more cost-effective intervention than IG1 and IG2, with greater detection of HPV infections, epithelial lesions and cancer than other strategies, albeit at a greater cost.

Trial Registration: Clinical Trials.gov Identifier NCT01373723

Background

Cancer of the cervix is the second most frequent cancer in the world among women, with at least 400,000 new cases being detected every year [1]. Despite being one of the neoplasms with an easy preventive and therapeutic approach, it remains an important cause of morbid-mortality, although the figures in developed countries are lower than those in developing countries. In Spain, the incidence of cervical cancer is 7.1%, with a mortality of 3.1 cases per 100,000 women/year and showing a progressive trend [2]. In the Department of Pathologic Anatomy of our reference hospital C. H. Parc Taulí, 18 cases of invasive cervical cancer and 464 pre-malignant lesions (ASCUS, AGUS, LSIL, HSIL) have been diagnosed corresponding to women referred to our sexual and reproductive primary health care services (SRHC) from 2003 to 2008.

The aetiological cause of cervical cancer is infection by the human papilloma virus (HPV) [3]. Seroptypes 16 and 18 are the most prevalent types in our setting and, together with serotypes 45, 31, 33, 52, 58 and 35, are responsible for 90% of cervical cancer, with a global prevalence of 99.7% of cases[4,5]. The systematic vaccination of 12-year-old girls initiated in 2008 may have an important impact on the prevalence of HPV within 15 to 20 years, with a potential reduction of 67 to 71% of

* Correspondence: amelia.acera@gmail.com
[1]Atenció a la Salut Sexual i Reproductiva (ASSIR) SAP Cerdanyola -Ripollet. Institut Catala de la Salut. Barcelona, Spain
Full list of author information is available at the end of the article

© 2011 Acera et al; licensee BioMed Central Ltd. This is an Open Access article distributed under the terms of the Creative Commons Attribution License (http://creativecommons.org/licenses/by/2.0), which permits unrestricted use, distribution, and reproduction in any medium, provided the original work is properly cited.

this virus [6]. High and continuous coverage of this vaccine in girls from 11 to 14 years of age may lead to a redefinition of screening, a variation in the schedules and, perhaps, the incorporation of primary screening with the HPV detection test in vaccinated women [7].

The Papanicolau cytology test introduced in the 1960s has allowed a reduction of up to 80% in the mortality by this disease with early diagnosis of precancerous lesions [8]. Thus, this continues to be the main diagnostic test used in screening programmes worldwide. One of the characteristics of the cytology test is its low sensitivity requiring periodical repetition. The test for the determination of HPV has recently been included in the screening programmes and, since 2006, 213 HPV positive cases have been detected in the study area.

In Catalonia, the protocol, which was revised and modified by the Oncology Management Director Plan and the Catalan Institute of Oncology in 2006, did incorporat the establishment of triennial periodicity of cytologies in women from 25 to 65 years of age as well as the incorporation of the HPV test in women from 40 to 65 years of age with no prior cytology within the previous 5 years or with a cytology carried out for longer than 5 years, abnormal cytology (no specified atypical squamous lesions) and women with post-conization control of intraepithelial lesions. The protocol also emphasizes the preventive role of the Basic Health Care Area (BHCA) and incorporates the figure of the midwife as a skilled professional for sample obtainment [9].

The research team decided to establish the age range for the population study between 30 to 70 years. The justification for the lower age limit is based on: 1.- although the second decade of life is when the greatest peak of infection by HPV is produced, the probability of its persistence is lower in young women, since the infection disappears in 90% of the cases after two years of follow up [7,10] 2.- early actions increase unnecessary interventions, generating anxiety in the woman and a work and economic overload on the public health care system [11]. 3.- establishing a limit after 30 years of age will detect the lesions derived from the persistence of HPV which, with treatment, will preserve fertility and avoid the evolution of the lesion towards invasive cancer [12].

In addition, the justification of the upper age limit is based on: 1.- recommendations of the Spanish Consensus for secondary prevention of cancer of the cervix in 2006 [13]. 2.- resolution num. 287 of the European Council (June 10, 2008 Luxemburg), related to the volume of cancer cases in the European Union and the mechanisms to reduce them [14]. 3.- an increase observed in the incidence of cancer in women born in the decade from 1930 to 1940 [2,13,14].

Eighty percent of the cases of cervical cancer in Catalonia have not undergone previous cytology during the 10 years prior to diagnosis [15]. Thus, an increase in screening coverage should be a priority objective for health care authorities if cervical cancer cases are to be reduced and women who do not periodically undergo cytology are to be identified. Some authors have reported that factors such as ethnic origin, age, education, and the socioeconomic levels condition participation in screening programmes [16]. Reasons to justify why women do not attend screening could include: (a) the perception of vulnerability; (b) the benefits perceived of screening; (c) anxiety; (d) bothersome; (e) fear of cancer; ans, (f) familial difficulties or personal circumstances [16,17]. With regard to the factors related to the health care system which influence the participation in screening in our setting, some authors have suggested: the absence of populational programmes, low sensitization with respect to preventive attitudes in cohorts of women of advanced age and the therapeutic health care overload in the primary care centres (PCC) [15].

The opportunistic preventive screening program in the Catalonian public health care system is carried out by the Sexual and Reproductive Health Care (SRHC) service located in the PCC and BHCA. The coverage of women attending our health care system (CIH) in 2008 (41.6%, figure obtained from the Systems of Primary Health Care Information (SPHCI) of the study setting) was similar to the one remaining at the SRHC of Catalonia.

A systematic review of the Cochrane collaboration [16], which evaluates interventions to stimulate the participation of women in the screening of this disease, concluded that invitations and educational interventions seem to be the most effective methods to increase absolute participation in the screening, although, to date, this has not been analysed from the point of view of efficiency.

At present, no study has evaluated from the efficiency point of view these interventions, though economic evaluation has been highly recommended for the cervical cancer screening programmes and HPV infections [18-20]. One key question influencing participation in screening programmes has been precision of population registers if an invitation letter is used (i.e. in some studies from 20 to 30% of the invited women were lost due to incorrect contact information) [21].

Due to the high prevalence of cervical cancer cases in this particular county we propose to launch a comparison of three different alternatives to improve the present coverage of the populational screening programmes in all the BHCA, therefore facilitating accessibility of population to the public health care system. The screening model proposed is centred on the recruitment of women with incorrect screening, performing cytology and the hybrid capture test for HPV to add diagnostic resolution due to the greater sensitivity of the test and

the absence of screening in this population of women. Hence, systematic screening should be incorporated since these women have a greater risk of having cervical disease because of not having visited the health care system before. Therefore, this would facilitate earlier action in detecting pre-malignant lesions, helpingto t reduce the incidence of invasive cancer. To achieve so, the research team propose three different interventions, consisting in sending: (a) an invitation letter to participate in the screening; (b) an invitation letter and an informative leaflet; and, (c) an invitation letter, an informative leaflet and a phone call reminder. Therefore, there is: one common action in the three different interventions, which has been scientifically validated as effective, consisting in a personalized invitation letter sent by the primary health care professionals including a fixed appointment to undergo through a cytology test and, other two different interventions (informative leaflet and reminder call) to evaluate approaches for which there are few studies assessing the effectiveness of attendance to screening programmes. Following the indications of the National Health Care System to optimise the interventions of cancer prevention [18], the aim is to assess which is the most cost-effective intervention for a cervical cancer screening programme.

Method/Design

An economic evaluation of three populational screening strategies for cervical cancer will be performed. (Figure 1, 2) Particularly, a cost-effectiveness analysis will be conducted. These interventions will be compared to the current opportunistic screening strategy using data of the multicentre randomised trial (CRICERVA). The analysis will be conducted from the National Health Care System perspective [22].

Design

Pragmatic, blinded, multicentre, randomised, controlled clinical trial with four branches, and a three years follow-up. The randomisation unit was BHCA.

Setting

Primary Health Care Services (PCS) of Cerdanyola-Ripollet, province of Barcelona, comprising 4

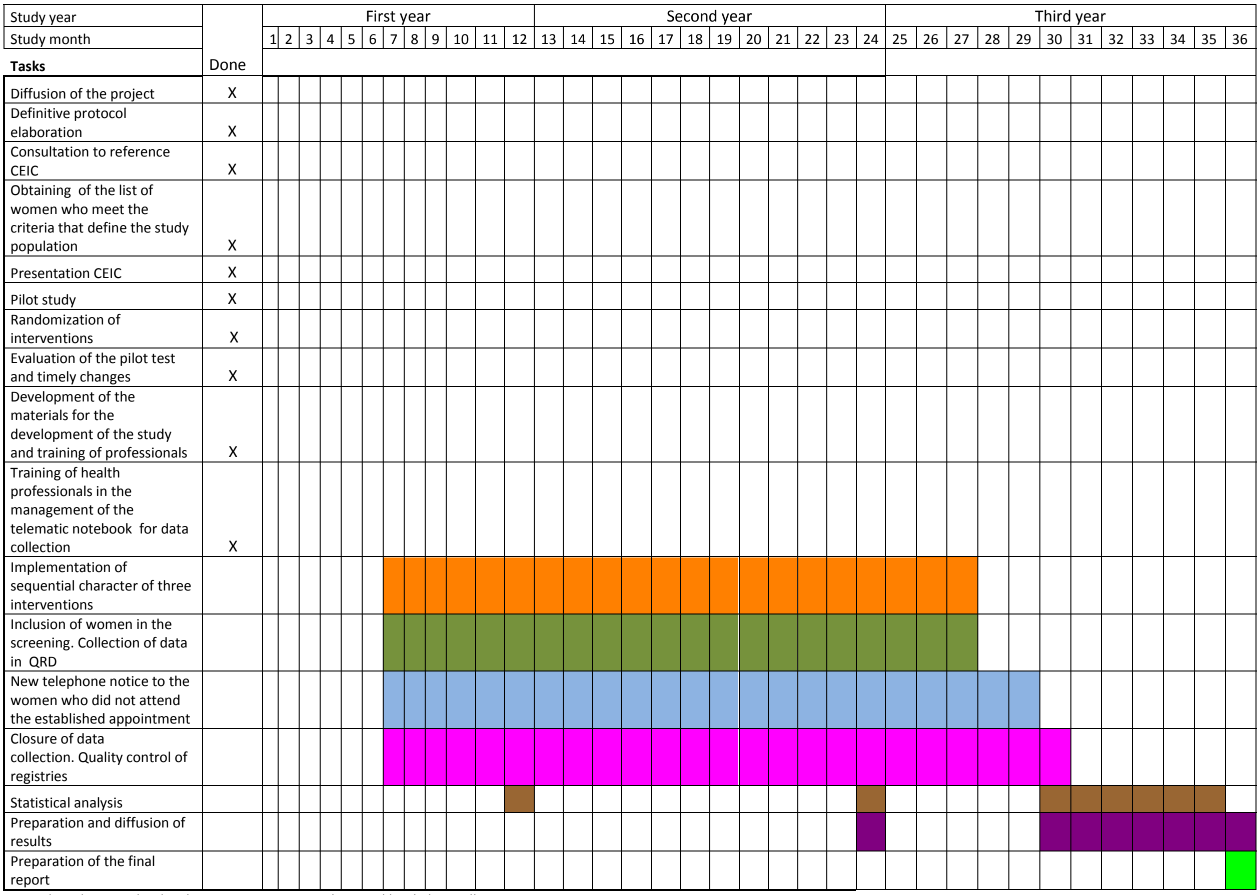

CEIC: Clinical Research Ethical Committees; **QRD**: Online workbook data collection

Figure 1 Timing of the Project.

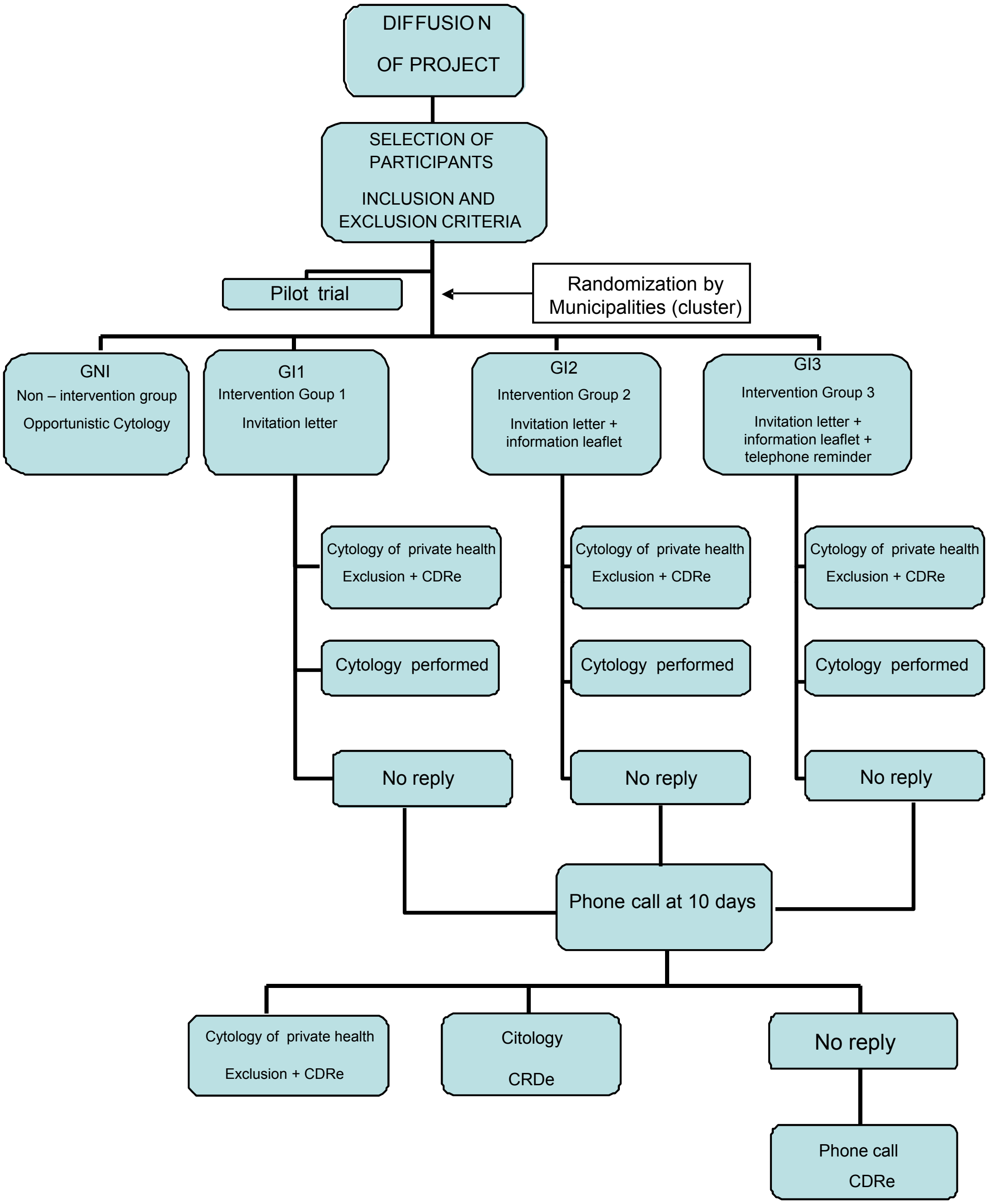

Figure 2 Study Algorithm.

municipalities and 5 BHCA. The population covered by this PCS is 120,293 inhabitants over 14 years. As there are four study groups and 5 BHCAs, only 4 BHCAs with most homogeneous socioeconomic criteria will be considered.

Population

20,994 women from 30 to 70 years of age with incorrect screening criteria (data obtained from SPHCI) ascribed to the BHCA will be included in the study. Incorrect screening will be defined as [9]: 1.- No cytology in the last 3 years from women between 30 to 40 years, 2.- No cytology in the previous 5 years from women between 40 to 65 years, 3.- No previous cytology history for females older than 65 years or women who have not had their last cytology before the age of 60. The exclusion criteria will be: (a) hysterectomised women, with a current history of pre-malignant lesions (AGUS,

ASCUS, LSIL, HSIL), carcinoma in situ and cervical-uterine cancer, HIV positive or other causes of immuno-suppression (since these women follow a specific protocol); (b) those residing outside the study setting for more than 6 months; and (c) those ascribed to the study BHCA but with a physician assigned in an UBA of another zone different from the one considered in the study.

Sample size

The sample size has been calculated based on the detection of a difference in effectiveness compared with the non intervention group (NIG). It has been calculated by multiplying the size of a simple randomised design by the design effect or factor of inflation. For the simple randomised design, on accepting an alpha risk of 0.05 and a beta risk of 0.20 in a bilateral contrast, 59 subjects will be required in the first group and 59 in the second group to detect a difference greater than or equal to 28.4% in the screening coverage of the 41.6% in the NIG. The lost to follow up rate has been estimated at 20%. The calculation of the sample has been performed with the Granmo 5.2 computer programme for Windows. According to a review of the literature [23-25], considering an intraclass correlation coefficient of 0.05 and a mean number of 3,500 women from 30 to 70 years of age with incorrect screening by BHCA, the design effect will be 176 and thus, 20,768 women with incorrect screening will be required.

Outcomes measures and statistical analysis

- *Decision analysis*

A Markov model will be developed to simulate the natural history of cervical-uterine cancer and to measure the impact, on costs and outcomes, of three different screening strategies. This same model of analytical decision has been used in several screening studies for cervical cancer [26-34]. The parameters of this model, clinical variables and costs, which would be included, are shown in the following sections of this protocol [35,36]. The population will be distributed across different health states of the model using transition probabilities over time. The temporal cycles of the Markov model will be 6 months of length during which time the state of health of the women may change or remain the same. These probabilities are conditioned to age, state of the HPV and history of the disease. The 11 states of health considered will be: healthy women, HPV infection, low grade intraepithelial lesion (L-SIL), high grade epithelial lesion (H-SIL), FIGO EIa stage invasive cancer (FIGOEla), FIGO EIb stage invasive cancer (FIGEIb), FIGO EIIa stage invasive cancer (FIGOEIIa), FIGO ELLb stage invasive cancer (FIGOEIIb), FIGO EIII stage invasive cancer (FIGOEIII), FIGO EIV stage invasive cancer (FIGOEIV), and death. These health states, recommended by the clinical experts of this study, are consistent with those extensively reported in the economic literature [26-34].

- *Clinical effectiveness measures*

The main effectiveness measures of this evaluation would be the total number of cytologies performed, HPV infections detected, lesions of different grades detected, episodes of cancer detected and the number of deaths avoided. The efficacy of the interventions will be obtained from the CRICERVA clinical trial.

- *Costs measures*

Micro-costing techniques will be used to estimate the health care costs of the different screening intervention of cervical cancer and treatments of different health states [37]. The costs will be presented in 2013 Euros (€) (year foreseen to finalize the study). Primary data of CRICERVA clinical trial will be used whenever possible. The costs will include the costs of: diagnosis, interventions and treatment for all women. The costs of diagnosis will include the cost of cytology, the HPV determination test and the control visit by the midwife or gynaecologist. The cost of the interventions will cover the cost of a full-time administrator, the personalized screening invitation letters, the informative leaflets and phone calls. The cost of treatment will depend on the state of health of the women but may include the costs of the visits to the gynaecologist, cytology tests, HPV determination, control visits with the physician, costs of radical hysterectomy, radiotherapy, chemotherapy, etc.

- *Temporal horizon*

The temporal horizon of the analysis will be the lifetime period of the woman.

- *Discount rate*

The discount rate to calculate costs and clinical outcomes will be 3% [22].

- *Cost-effectiveness analysis*

The cost-effectiveness analysis will be conducted comparing costs and effectiveness, measured in natural units, of the different alternatives to usual practice. In most cases the data will be obtained from the CRICERVA clinical study. The comparison will be performed using the Incremental Cost-Effectiveness Ratio (ICER) defined as the ratio of the difference in costs and the difference in effectiveness. The results will be expressed in terms of €/per unit of effectiveness.

- *Uncertainty*

To study the uncertainty, an univariate and multivariate deterministic sensitivity analysis will be undertaken of the relevant parameters considered.

- *Statistical analysis*

All the statistical analyses will be performed using the SPSS v.18 programme and Microsoft Excel. The

economic evaluation will be carried out according to the principle to be determined. Descriptive analysis of each of the groups will be performed. The mean values and standard deviations of the costs and the corresponding intervals of confidence at 95% will be presented. The final result will be reported in terms of effectiveness, cost and ICERs for each of the alternatives compared.

Ethical Aspects

The investigators are committed to respect the prevailing norms of Good Clinical Practice as well as the requisites of the Declaration of Helsinki and the clauses of general and particular ethical conditions related to the right to privacy, anonymity and confidentiality. Neither the first name nor surname or any other type of data indicating the identification of the women will be registered. Therefore, identification will be made by numeric codes. Since this type of study is developed in the usual clinical setting, authorisation and support must be and has already been granted by the representatives and authorities of the collectives involved and thus, individualised informed consent is not necessary. Nonetheless, the research team decided that women attending the consultation for the cytology should sign the consent form. The protocol has been evaluated by the ECCI of the Jordi Gol IIPC.

Limitations

Randomisation by groups will avoid the potential introduction of selection bias which may be produced among the interventions performed at the same site. Since the characteristics of the study do not allow the application of the double-blind masking technique, the masked response evaluation will be used to ensure that the measurement and interpretation of the dependent variables is carried out the same way in all groups. The possible loss of information, which may be produced in women doing screening outside public health care if they are not contacted by the research team, will be minimised with a phone call reminder. This will be made when the women do not attend the appointment. The language difficulties in women from other countries will be solved with cultural mediators at each site. Within the setting of the study, the administrative personnel have been updating the postal addresses of the users attending the BHCA since 2007 and, therefore the postal registry is quite precise, thereby reducing the potential loss of letters.

Abbreviations

(AETM): Agency for Evaluation of Technology and Medical Investigation; **(BHCA)**: Basic Health Care Area; **(AGUS):** Atypical glandular cells of undetermined significance; **(ASCUS):** Atypical squamous cells of undetermined significance; **(PCC):** Primary Care Centre; **(ECCI):** Ethical Committee of Clinical Investigation; **(eCCN):** Electronic Data Collection Notebook; **(PCT):** Primary Care Team; **(c-CH):** Computerised Clinical History; **(IT):** Investigative Team; **(IG):** Intervention Group; **(NIG):** Non intervention Group; **(HC2):** Hybrid Capture 2; **(HSIL):** High grade Squamous Intraepithelial Lesion; **(CIO):** Catalan Institute of Oncology; **(CIH):** Catalan Institute of Health; **(IIPC):** Institute of Investigation in Primary Care; **(LSIL):** Low grade Squamous Intraepithelial Lesion; **(SRHC):** Sexual and Reproductive Health Care; **(PCS):** Primary Care Service; **(ISU):** Investigation Support Unit; **(HSV):** Herpes Simple Virus; **(HIV):** Human Immunodeficiency Virus; **(HPV):** Human Papilloma Virus; **(UBA):** Unitat Bàsica Assistencial (General Practicioner and Nurse Team).

Acknowledgements and financial support

Josep Mª Bonet, Gemma Falguera, Dolors Costa, for their institutional support.
Silvia Sanjose, for her willingness to attend our consultations.
Maribel Usart and Javier Arribas, for their contribution in improving patient management and follow up.
This protocol has received financial Grant from the Fondo de Investigación Sanitaria del Instituto Carlos III de Madrid (exp PI10/01275).

Author details

[1]Atenció a la Salut Sexual i Reproductiva (ASSIR) SAP Cerdanyola -Ripollet. Institut Catala de la Salut. Barcelona, Spain. [2]Health Economics Research Group, Brunel University, UK. [3]SAP Cerdanyola-Ripollet. Institut Català de la Salut. Barcelona, Spain. [4]Unitat de Suport a la Recerca Metropolitana Nord. IDIAP Jordi Gol. Sabadell, Spain. [5]Sistemes d'Informació Sanitària del SAP Cerdanyola-Ripollet. Institut Català de la Salut. Barcelona, Spain. [6]Gerencia de Atención Primaria de Ávila. Ávila, Spain. [7]Departament d'Infermeria. Universitat Autònoma de Barcelona. Bellaterra, Spain.

Authors' contributions

AA and GP formulated the study question. AA, AR, PS, PH, JMB, GP and JMM participed in the revision of the bibliography and design of the study methodology. AA, AR, PS, PH, JMB, GP, JMM, MTB, NS and PT collaborated carrying out the study. AA, AR, PS, PH, JMB, GP, JMM, MTB, NS and PT contributed in the writing and preparation of the present manuscript. All of the authors have read and approved of the present manuscript.

Competing interests

The authors declare that they have no competing interests.

Received: 22 June 2011 Accepted: 19 October 2011
Published: 19 October 2011

References

1. Maxwell D, Bray F: **Magnitud de los cánceres atribuibles al VPH.** *Vaccine de* 2006, **24(Sup 3)**:11-24.
2. Bosch FX, Castellsagué X, Cortés J, Puig-Tintoré LM, Roura E, de Sanjose S, Torné A: **Estudio AFRODITA: cribado del cáncer de cuello uterino en España y factores relacionados.** Madrid: Edición patrocinada por GSK; 2009.
3. Muñoz N, Castellsagué X, de González AB, Gissmann L: **HPV in the etiology of human cancer.** *Vaccine de* 2006, **24(Sup 3)**:1-10.
4. Walboomers JM, Jacobs MV, Manos MM, Bosch FX, Kummer JA, Shah KV, Snijders PJ, Peto J, Meijer CJ, Muñoz N: **Human papillomavirus is a necessary cause of invasive cervical cancer worldwide.** *J Clin Pathol* 1999, **189(1)**:12-9.
5. Cortés J, Alba A, Andía D, Ramón y Cajal JM, Velasco J, Vilaplana E: **Interacción vacuna frente al VPH/cribado del Cáncer de Cuello de Útero.** *Actas del Primer Foro Español sobre el Virus del Papiloma Humano: 13-14 de junio de 2008* Sevilla. Madrid: Sanofi Pasteur MSD; 2010.
6. De Sanjosé S: **The value of Pap screening after the introduction of HPV vaccines.** *Actas de la Segunda Jornada Internacional sobre virus del papiloma humano y cáncer: 29 de enero de 2009* Barcelona. Barcelona: GlaxoSmithKline; 2009.
7. Wright TC, Bosch FX, Franco EL, Cuzick J, Schiller JT, Garnett GP, Meheus A: **Las vacunas contra el VPH y el cribado en la prevención de cáncer de cuello uterino: Conclusiones de una reunión internacional de expertos celebrada en el año 2006.** *Vaccine de* 2006, **24(Sup 3)**:279-290.

8. Kitchener HC, Castle PE, Cox JT: **Logros y limitaciones del cribado citológico cervical.** *Vaccine de* 2006, **24(Sup 3)**:67-75.
9. **Protocolo de las actividades para el cribado del cáncer de cuello uterino en Atención Primaria (monografía en internet).** Barcelona: Generalitat de Catalunya, Departament o de Salud; 2006 [http://www.gencat.cat/salut/depsalut/pdf/collut.pdf], (citado el 15 de noviembre de 2008). Disponible en.
10. Moscicki AB, Schiffman M, Kjaer S, Villa LL: **Avances recientes en la historia natural del VPH y del cáncer anogenital.** *Vaccine de* 2006, **24(Sup 3)**:43-53.
11. Clare J, Edwards D, Bagnall H, Pearmain P, Lawrence G: **The use of cervical screening history data to interpret cervical cancer incidence trends.** *Journal of Public Health* 2008, **30(2)**:171-7.
12. Díaz M: **Challenges of HPV Vaccination.** *Proceeding of the Workshop on Human Papillomavirus (HPV) and Cancer: 29 de enero de 2009* Barcelona. Barcelona: GlaxoSmithKline; 2009.
13. Oncoguía SEGO: **Cáncer de Cuello Uterino 2008.** *Guías de práctica clínica en cáncer ginecológico i mamario* Publicaciones SEGO; 2008.
14. **Guidance for the introduction of hpv vaccines in European Union countries.** *Stockholm* European CDC (ECDC); 2008, http://ecdc.europa.eu/en/publications/Publications/0801_GUI_Introduction_of_HPV_Vaccines_in_EU.pdf .
15. **Protocolo para mejorar la cobertura del cribado de cáncer de cuello de útero a partir de la red asistencial pública en Cataluña.** *Newsletter on Human Papillomavirus* 2008, **4-5[http://www.hpvtoday.com Suplemento en español n° 3], Disponible en.**
16. Forbes C, Jepson R, Martin-Hirsch P: **Intervenciones para estimular la participación de las mujeres en el cribaje de cáncer cervicouterino (Revisión Cochrane traducida).** Chichester, UK: John Wiley & Sons, Ltd; 2008, **3[http://www.bibliotecacochrane.com/BCPGetDocument.asp?SessionID= 2407697&DocumentID=CD002834]], En: La Biblioteca Cochrane Plus, 2008 Número 4. Oxford: Update Software Ltd..**
17. Eaker S, Adami HO, Granath F, Wilander E, Spare'n P: **A large population-based randomized controlled trial to increase attendance at screening for cervical cancer.** *Cancer Epidemiol Biomardkers Prev* 2004, **13(3)**:346-54.
18. Bosch FX, de Sanjosé S, Miralles C: **Castellsagué X La prevención del pre-cáncer i del cáncer cervical en España: nuevas opciones para el siglo XXI.**[http://www.edicionesmayo.es], Folia clínica en Obstetricia Ginecología 2010 (n° 81). Disponible en.
19. Cuzick J, Sasieni P, Davies P, Adams J, Normand C, Frater A, Van Ballegooijen M, Van den Akker-van Marle E: **Assessing effectiveness, costs and cost-effectiveness of cervical cancer screening and HPV testing (Chapter 7). In: A systematic review of the role of human papillomavirus testing within a cervical screening programme.** *Health Technology Assessment* 1999, **3(14)**:95-123.
20. Mansley EC, McKenna MT: **Importance of perspective in economic analyses of cancer screening decisions.** *The Lancet* 2001, **358**:1169-73.
21. Rossi PG, Esposito G, Brezzi S, Brachini A, Raggi P, Federici A: **Estimation of Pap-test coverage an area with am organized screening.** *BMC Health services research* 2006, **6**:36-46.
22. Lopez Bastida J, Oliva J, Antonanzas F, Garcia-Altes A, Gisbert R, Mar J, Puig-Junoy J: **Propuesta de guía para la evaluación económica aplicada a las tecnologías sanitarias.** *Gaceta Sanitaria* 2009.
23. Adams G, Gulliford MC, Ukoumunne OC, Eldridge S, Chinn S, Campbell MJ: **Patterns of intra-cluster correlation from primary care research to inform study design and analysis.** *J Clin Epidemiol* 2004, **57**:785-794.
24. Parker DR, Evangelou E, Eaton CB: **Intraclass correlation coefficients for cluster randomized trials in primary care: the cholesterol education and research trial (CEART).** *Contemp Clin Trials* 2005, **26**:260-267.
25. Ukoumunne OC, Gulliford MC, Chinn S, Sterne JA, Burney PG: **Methods for evaluating area-wide and organisation-based interventions in health and health care: a systematic review.** *Health Technol Assess* 1999, **3(5)**:iii-92.
26. Goldie SJ, Gaffikin L, Goldhaber-Fiebert JD, Gordillo-Tobar A, Levin C, Mahé C, Wright TC: **Cost-effectiveness of Cervical-Cancer Screening in Five Developing Countries.** *The New England Journal of Medicine* 2005, **353**:2158-68.
27. Siebert U, Sroczynski G, Hillemanns P, Engel J, Stabenow R, Stegmaier C, Voigt K, Gibis B, Hölzel D, Goldie SJ: **The German Cervical Cancer Screening Model: development and validation of a decision-analytic model for cervical cancer screening in Germany.** *European Journal of Public Health* 2006, **16(2)**:185-92.
28. Mandelblatt JS, Lawrence WF, Mizell Womack S, Jacobson D, Yi B, Hwang Y, Gold K, Barter J, Shah K: **Benefits and Costs of Using HPV Testing to Screen for Cervical Cancer.** *JAMA* 2002, **287(18)**:2372-81.
29. Goldie S, Kuhn L, Denny L, Pollack A, Wright TC: **Policy Analysis of Cervical Cancer Screening Strategies in Low-Resource Settings.** *JAMA* 2001, **285(24)**:3107-15.
30. Berkhof J, de Bruijne MC, Zielinski GD, Bulkmans NWJ, Rozendaal L, Snijders PJF, Verheijen RH, Meijer CJ: **Evaluation of cervical screening strategies with adjunct high-risk human papillomavirus testing for women with borderline or mild dyskaryosis.** *International Journal of cancer* 2006, **118**:1759-68.
31. Mittendorf T, Petry KU, Iftner T, Greiner W, von der Schulenburg JM: **Economic evaluation of human papillomavirus screening in Germany.** *European Journal of Health Economics* 2003, **4(3)**:209-15.
32. Vijayaraghavan A, Efrusy M, Lindeque G, Dreyer G, Santas C: **Cost effectiveness of high-risk HPV DNA testing for cervical cancer screening in South Africa.** *Gynecologic Oncology* 2009, **112(2)**:377-83.
33. Kim JJ, Wright TC, Goldie SJ: **Cost-effectiveness of Alternative Triage Strategies for Atypical Squamous Cells of Undetermined Significance.** *JAMA* 2002, **287(18)**:2382-90.
34. Holmes J, Hemmett L, Garfield S: **The cost-effectiveness of human papillomavirus screening for cervical cancer. A review of recent modeling studies.** *European Journal of Health Economics* 2005, **6(1)**:30-7.
35. Briggs A, Sculpher M: **An introduction to Markov modelling for economic evaluation.** *Pharmacoeconomics* 1998, **13(4)**:397-409.
36. Karnon J, Brown J: **Selecting a decision model for economic evaluation: a case study and review.** *Health Care Management Science* 1998, **1**:133-140.
37. Drummond MF, Sculpher MJ, Torrance GW, O'Brien BJ, Stoddart GL: **Methods for the economic evaluation of health care programmes.** Oxford university Press; 2005.

Pre-publication history

The pre-publication history for this paper can be accessed here:
http://www.biomedcentral.com/1472-6963/11/278/prepub

doi:10.1186/1472-6963-11-278
Cite this article as: Acera *et al.*: Economic evaluation of three populational screening strategies for cervical cancer in the county of Valles Occidental: CRICERVA clinical trial. *BMC Health Services Research* 2011 **11**:278.

Submit your next manuscript to BioMed Central and take full advantage of:

- **Convenient online submission**
- **Thorough peer review**
- **No space constraints or color figure charges**
- **Immediate publication on acceptance**
- **Inclusion in PubMed, CAS, Scopus and Google Scholar**
- **Research which is freely available for redistribution**

Submit your manuscript at
www.biomedcentral.com/submit

BioMed Central
