# Supplementary material for: Increasing Cervical Cancer Screening Coverage: A Randomised, Community-Based Clinical Trial
Source: PLoS One. 2017 Jan 24;12(1):e0170371. doi: 10.1371/journal.pone.0170371 (PMC5261609; doi:10.1371/journal.pone.0170371)
Supplement: S2 File — (PDF) [file pone.0170371.s002.pdf]

MEMORIA DE SOLICITUD DEL PROYECTO DE INVESTIGACIÓN EN SALUD

Expediente nº

PI10/01275

TÍTULO:

Evaluación Económica de tres estrategias de cribado poblacional de cáncer de cérvix en la comarca del Valles Occidental: Estudio CRICERVA

Investigador/a principal: AMELIA ACERA PEREZ

TIPO DE PROYECTO: INDIVIDUAL ☒ COORDINADO ☐ MULTICÉNTRICO ☐

NOMBRE COORDINADOR/A (COORDINADOS O MULTICÉNTRICOS DE VARIAS SOLICITUDES):

DURACIÓN: 3 años ☒

RESUMEN (objetivos y metodología del proyecto): *(Máximo 250 palabras)*

Contexto: Un elevado porcentaje de los casos de cáncer cervical no tienen citología previa durante los 10 años anteriores al diagnóstico. Diferentes intervenciones poblacionales pueden mejorar la cobertura en el sistema público, aunque también aumentar los costes. *Objetivo: Comparar la efectividad y los costes de tres tipos de intervenciones de carácter poblacional para aumentar el número de participantes en los programas de cribado de cáncer de cuello uterino dirigidas desde Atención Primária a las mujeres de cuatro áreas básicas de salud *Métodos: Se realizará un análisis coste-efectividad desde la perspectiva del Sistema Público de Salud. Mujeres (20.994) con criterios de cribado incorrecto de cuatro áreas de salud básica del Vallés Occidental, Barcelona. Las pacientes serán distribuidas aleatoriamente en el grupo control y los tres grupos de intervenciones (GI1: carta de invitación para participar en el cribado; GI2: carta invitación y folleto informativo; GI3: carta invitación, folleto informativo y llamada recordatorio), y seguidas a lo largo de tres años. La medida de efectividad clínica será nº de infecciones de VPH, lesiones epiteliales y cáncer de cuello uterino. Medidas secundárias de efectividad será el número de muertes evitadas. Se llevarà a cabo un análisis de sensibilidad univariante y multivariante .*Resultados esperados: Se espera que la GI3 sea una estratégia más coste-efectiva que GI1 y GI2, generando mayores detecciones de infecciones de VPH, lesiones epitaliales y cáncer que las demás estratégias, aunque a mayor coste. Se preveé que el ratio coste-efectividad esté por debajo del umbral de los 20.000€, y por tanto recomendar GI3 como estratégia poblacional desde el punto de vista de la eficiencia.

TITLE:

Economic Evaluation of a poblational cervix cancer screening program: the CRICERVA study

SUMMARY (objectives and methodology):

Background: A high percentage of cervical cancer cases have not had any cytology test for the last ten years prior to diagnosis. Different population interventions could improve the population coverage of screening, though it could also lead to a rise in costs for the health system. *Objective: To compare costs and effectiveness of three different population interventions designed to increase the number of participants in the cervical cancer screening programmes, lead by Primary Care centres for women living in four different basic health areas. *Methods: To conduct a cost-effectiveness analysis from the National Health System perspective. Women (20.994) with incorrect screening criteria of four different health areas in Vallés Occidental, Barcelona were recruited. Patients were randomly distributed to the control and three interventions groups: (GI1: invitation letter to participate in the screening program; GI2: invitation letter and informative leaflet; GI3: invitation letter, informative leaflet and reminding call), and will be followed for three years. The effectiveness measure will be number of infections of HPV, epithelial lesions and cervical cancer episodes. Secondary effectiveness measures would be number of deaths avoided. A univariate and multivariate deterministic sensitivity analysis will be carried out.*Expected results: It is expected that group intervention 3 (GI3) will be more cost-effective compared to GI1 and GI2, detecting a higher number of HPV infections, epithelial lesions and cervical cancer episodes, though it might [illegible]

MEMORIA DE SOLICITUD DEL PROYECTO DE INVESTIGACIÓN

Expediente nº

PI10/01275

Investigador/a principal: AMELIA ACERA PEREZ

Antecedentes y estado actual del tema (citar las referencias incluidas en el apartado siguiente).
(Máximo 3 páginas)

En el anexo 1 se especifican las abreviaturas que se utilizarán a lo largo del texto.

El cáncer de cuello uterino es el segundo cáncer más frecuente en el mundo entre las mujeres detectándose al menos 400.000 casos nuevos cada año (1). A pesar de ser una de las neoplasias de más fácil abordaje preventivo y terapéutico todavía es una causa importante de morbi-mortalidad entre las mujeres, aunque las cifras para los países desarrollados son inferiores que las de los países en vías de desarrollo. En España, el cáncer de cérvix tiene una incidencia de 7,1 y una mortalidad de 3,1 casos por 100.000 mujeres/año, con tendencia progresiva (2). En el servicio de Anatomía Patológica del C. H. Parc Taulí, nuestro hospital de☐ referencia, han diagnosticado 18 casos de cáncer invasivo de cuello uterino y 464 lesiones pre-malignas (ASCUS, AGUS, LSIL, HSIL) correspondientes a mujeres derivadas por nuestro servicio (ASSIR) entre 2003 y 2008.

La causa etiológica del cáncer de cuello uterino es la infección del Papilomavirus Humano (VPH) (3). Los serotipos 16 y el 18 son los más prevalentes en nuestro entorno y responsables junto con el 45, 31 ,33, 52, 58 y 35 de aproximadamente el 90% de cánceres de cuello uterino, siendo la prevalencia global del 99,7% (4, 5). La vacunación sistemática iniciada el año 2008 a niñas de 12 años puede suponer un impacto importante en la prevalencia del VPH en un plazo de 15 a 20 años, pudiendo ser la reducción potencial de la misma de un 67 a un 71% (6). Una cobertura alta y continuada de esta vacuna para las niñas entre 11 y 14 años, puede hacer redefinir el cribado, variar las pautas y quizá incorporar como cribado primario en las mujeres vacunadas la prueba de detección del VPH en un futuro (7).

La citología de Papanicolau introducida en los años 60 ha permitido reducir la mortalidad por esta enfermedad hasta un 80% al permitir un diagnóstico precoz de lesiones precancerosas (8). Por ello, sigue siendo la principal prueba diagnóstica utilizada en los programas de cribado a nivel mundial. Una de las características de la citología es su baja sensibilidad, lo que conlleva a su repetición periódicamente. Recientemente se ha incluido en los programas de cribado la prueba de determinación del VPH, y desde el 2006 hasta el momento se han detectado en la zona de estudio 213 casos positivos de VPH.

En Cataluña, el protocolo revisado y modificado en 2006 por el Plan Director de Oncología y el Instituto Catalán de Oncología, incorpora como novedades el establecimiento de la periodicidad trianual de las citologías en mujeres de 25 a 65 años, así como la incorporación del test del VPH en mujeres de 40 a 65 años sin citología previa en los cinco años anteriores, mujeres mayores de 65 años sin historia previa de citologías o con citología previa de más de cinco años, citología anormal (lesiones de atipia escamosa no especificada) y mujeres con control post-conización de lesiones intraepiteliales. El protocolo también hace hincapié en el papel preventivo de las Áreas Básicas de Salud (ABS) e incorpora la figura de la matrona como profesional capacitado para la obtención de la muestra (9).

El equipo investigador, en el presente estudio, ha decidido establecer el rango de edad de la población de estudio entre 30 y 70 años. La justificación del límite inferior de edad se basa en: 1.- Aunque es en la segunda década de la vida cuando se produce el mayor pico de infección por VPH, la probabilidad de persistencia del mismo (condición para el desarrollo de displasia) es menor en mujeres jóvenes ya que la infección desaparece en un 90% de los casos tras dos años de seguimiento

(7, 10) 2.- Actuaciones más precoces aumentan las intervenciones innecesarias, generando angustia en la mujer y una sobrecarga laboral y económica en el sistema sanitario público (11) . 3.- Estableciendo el límite a partir de los 30 años se detectarían las lesiones derivadas de la persistencia del VPH, que al ser tratadas preservarían la fertilidad y evitarían la evolución de la lesión hacia cáncer invasivo (12).

La justificación del límite superior de edad se basa en: 1.- Recomendaciones del Consenso Español del 2006 de prevención secundaria del cáncer de cuello uterino (13). 2.- Resolución 287 del consejo de Europa (10 de Junio de 2008 Luxemburgo), relativas al volumen del cáncer en la Unión Europea y los mecanismos para reducirlo (14). 3.- Así como en algunos estudios (2, 13, 14) en los que se ha observado un aumento de la incidencia de cáncer en mujeres nacidas en la década entre 1930 y 1940.

En Cataluña el 80% de los casos de cáncer cervical no tenían citología previa durante los 10 años anteriores al diagnóstico (15). Por tanto, aumentar la cobertura del cribado ha de ser objetivo prioritario de los responsables de las políticas sanitarias si se pretende disminuir los casos de cáncer de cuello uterino e identificar a aquellas mujeres que no se realizan periódicamente una citología. Algunos autores han definido que factores como el origen étnico, la edad, la educación y el nivel socioeconómico, condicionan la participación en los programas de cribado (16). Entre los motivos posibles por los cuales las mujeres no acuden al cribado se apuntan: la percepción de vulnerabilidad, los beneficios percibidos del cribado, la ansiedad, la perturbación, el temor al cáncer o las dificultades familiares o circunstancias personales (16). En cuanto a los factores relacionados con el sistema sanitario de nuestro entorno que influyen en la participación en el cribado, algunos autores sugieren: la ausencia de programas poblacionales, la baja sensibilización en cuanto a las actitudes preventivas en cohortes de mujeres de edad avanzada y la sobrecarga asistencial terapéutica en los centros de Atención Primaria (15).

El cribado oportunista preventivo en el sistema público en Cataluña se realiza en los servicios de Atención a la Salud Sexual y Reproductiva (ASSIR) ubicados en los CAP y ABS. La cobertura en el año 2008 en las mujeres que acuden a nuestro sistema sanitario (ICS) fue del 41,6% (cifra obtenida de los Sistemas de Información Sanitaria (SIS) del ámbito de estudio), similar a la del resto de ASSIR de Cataluña. Esta cobertura difiere de los resultados obtenidos en el estudio Afrodita que proporciona una cobertura del 80%.

En una revisión sistemática de la colaboración Cochrane (16), que evalúa las intervenciones para estimular la participación de las mujeres en el cribado de esta patología, los autores llegan a la conclusión de que las invitaciones e intervenciones educacionales parecen ser los métodos más efectivos para aumentar la participación absoluta en el cribado, aunque hasta ahora no se ha analizado desde el punto de vista de la eficiencia.

Actualmente no hay estudios que evalúen económicamente estas intervenciones para estimular la participación de las mujeres en este tipo de cribado, siendo muy recomendada esta evaluación económica para el cribado de cáncer de cuello uterino y de infecciones del VPH (19, 20). Una cuestión clave que influye en la participación de los programas de cribado, es la exactitud de los registros de la población si se utiliza la carta de invitación, ya que en algunos estudios, debido a detalles de contacto incorrectos, se han llegado a perder entre un 20 y un 30% de las mujeres convocadas (21).

Ante los casos de cáncer de cuello uterino invasivo diagnosticados en mujeres de nuestra zona y una cobertura actual del 41,6%, nos proponemos poner en marcha una comparacion de tres alternativas

diferentes para mejorar la actual cobertura de los programas de cribado poblacional en todas las Áreas Básicas de Salud de nuestro SAP, facilitando así la accesibilidad al sistema sanitario público. El modelo de cribado que se propone se centraría en el reclutamiento de las mujeres con un cribado incorrecto, realizando citologia y test de captura de híbridos para el VPH, para añadir resolución diagnóstica, debido a la mayor sensibilidad de la prueba y a la ausencia de cribado en esta población de mujeres. Posteriormente se incorporaràn al cribado sistemàtico, ya que son éstas mujeres quienes tienen mayor riesgo de tener patología de cuello uterino al no acudir al sistema sanitario. De esta forma se actuará precozmente sobre las lesiones pre-malignas y se disminuirá la incidencia del cáncer invasivo.

Basándonos en la evidencia existente sobre actuaciones efectivas para aumentar la cobertura del cribado de cuello uterino, el equipo investigador propone tres tipos de intervenciones: una intervención común en todos los grupos, avalada científicamente como efectiva, que consiste en una invitación por carta personalizada y remitida por los profesionales sanitarios de Atención Primaria que habitualmente atienden a la mujer, en la que se incluirá una cita fija para realizarse una citología; y otras dos para explorar intervenciones sobre las que no hay estudios suficientes que valoren la efectividad sobre participación informada y recordatorios de asistencia al cribado. Se pretende, siguiendo las indicaciones del Sistema Nacional de Salud para optimizar las intervenciones de prevención del cáncer (18) conocer cual de las intervenciones propuestas es la más coste-efectiva.

MEMORIA DE SOLICITUD DEL PROYECTO DE INVESTIGACIÓN

Expediente nº

PI10/01275

Investigador/a principal: AMELIA ACERA PEREZ

Bibliografía más relevante. *(Máximo 1 página)*

*1. Maxwell D, Bray F. Magnitud de los cánceres atribuibles al VPH. Vaccine. 2006;24 Suppl 3:S11-24. *2. Bosch FX, Castellsagué X, Cortés J, Puig-Tintoré LM, Roura E, de Sanjose S, Torné A. Estudio AFRODITA: cribado del cáncer de cuello uterino en España y factores relacionados. Madrid: Edición patrocinada por GSK; 2009. *3. Muñoz N, Castellsagué X, de González AB, Gissmann L. HPV in the etiology of human cancer. Vaccine. 2006;24 Suppl 3:S1-10. *4. Walboomers JM, Jacobs MV, Manos MM, Bosch FX, Kummer JA, Shah KV, et al. Human papillomavirus is a necessary cause of invasive cervical cancer worldwide. J Pathol. 1999;189(1):12-9. *5. Cortés J, Alba A, Andía D, Ramón y Cajal JM, Velasco J, Vilaplana E. Interacción vacuna frente al VPH/cribado del Cáncer de Cuello de Útero. Acta del 1er Foro Español sobre el Virus del Papiloma Humano; 2008 Jun 13-14; Sevilla, España. *6. De Sanjosé S. The value of Pap screening after the introduction of HPV vaccines. Acta de la II Jornada Internacional sobre virus del papiloma humano y cáncer; 2009 Enero 29; Barcelona, España. *7. Wright TC, Bosch FX, Franco EL, Cuzick J, Schiller JT, Garnett GP, et al. Las vacunas contra el VPH y el cribado en la prevención de cáncer de cuello uterino: Conclusiones de una reunión internacional de expertos celebrada en el año 2006. Vaccine. 2006;24 Suppl 3:S279-290. *8. Kitchener HC, Castle PE, Cox JT. Logros y limitaciones del cribado citológico cervical. Vaccine. 2006;24 Suppl 3: S67-75. *9. Generalitat de Catalunya. Departament de Salut. Protocolo de las actividades para el cribado del cáncer de cuello uterino en la Atención Primaria. Direcció General de Planificació i Avaluació. Barcelona, 2006. *10. Moscicki AB, Schiffman M, Kjaer S, Villa LL. Avances recientes en la historia natural del VPH y del cáncer anogenital. Vaccine. 2006;24 Suppl 3: S43-53. *11. Clare J, Edwards D, Bagnall H, Pearmain P, Lawrence G. The use of cervical screening history data to interpret cervical cancer incidence trends. Journal of Public Health. 2008;30(2):171-7. *12. Díaz M. Retos de la vacunación frente al VPH. Acta de la II Jornada Internacional sobre virus del papiloma humano y cáncer; 2009 Enero 29; Barcelona, España. *13. Recomendaciones del Consenso Español del 2006 de prevención secundaria del cáncer de cuello uterino. *14. Resolución 287 del consejo de Europa, (10 de Junio de 2008 Luxemburgo) relativas al volumen del cáncer en la Unión Europea y los mecanismos para reducirlo (http://www.register.consilium.europa.eu). *15. De Sanjosé S, Ibañez R, Piris M. Protocolo para mejorar la cobertura del cribado de cáncer de cuello de útero a partir de la red asistencial pública en Cataluña. HPV Today [serie en Internet]. 2008 Feb [citado 11 Nov 2008]. Disponible en: http://www.hpvtoday.com/hpvtoday-sus/pdf/HPV-Today_15_es.pdf. *16. Forbes C, Jepson R, Martin-Hirsch P. Intervenciones para estimular la participación de las mujeres en el cribaje de cáncer cervicouteino (Revisión Cochrane traducida). En: La Biblioteca Cochrane Plus, 2008 Número 2. Oxford: Update Software Ltd. Disponible en: http://www.update-software.com. *17. Eaker S, Adami HO, Granath F, Wilander E, Spare'n P. A large population-based randomized controlled trial to increase attendance at screening for cervical cancer. Cancer Epidemiol Biomardkers Prev. 2004;13(3):346-54. *18. Larizgoitia I. Diagnóstico precoz de cáncer cervical: conocimiento actual sobre viejas y nuevas tecnologías. Barcelona: Agència d'Avaluació de Tecnologia i Recerca Mèdiques. Servei Català de la Salut. Departament de Sanitat i Seguretat Social. Generalitat de Catalunya. Octubre 2001 (BR02/2001). *19. Cuzick J, Sasieni P, Davies P, Adams J, Normand C, Frater A, et al. Assessing effectiveness, costs and cost-effectiveness of cervical cancer screening and HPV testing (Chapter 7). In: A systematic review of the role of human papillomavirus testing within a cervical screening programme. Health Technology Assessment. 1999; 3(14):95-123. *20. Mansley EC, McKenna MT. Importance of perspective in economic analyses of cancer screening decisions. The Lancet. 2001;358:1169-73. *21. Rossi PG, Esposito G, Brezzi S, Brachini A, Raggi P, Federici A. Estimation of Pap-test coverage an area with am organized screening. BMC Health services research. 2006;6:36-46.

Expediente nº

PI10/01275

Investigador/a principal: AMELIA ACERA PEREZ

Hipótesis.

La estratégia de otorgar una carta de invitación, un folleto informativo y hacer una llamada recordatorio, aunque genera mayores costes, es la estrategia más coste-efectiva. a nivel poblacional, para el Sistema Público de Salud. Además, estas diferentes estratégias poblacionales, dirigidas desde Atención Primaria, permitirán aumentar la cobertura (41,6% actual) del cribado oportunista.

Objetivos.

Objetivo Principal:

Evaluación Económica de tres tipos de intervenciones de carácter poblacional dirigidas desde Atención Primaria para mejorar la cobertura en el sistema público de salud del cribado de cáncer de cuello uterino de las mujeres entre 30 y 70 años con criterios de cribado incorrecto.

Objetivos Secundarios:

1.- Evaluar la efectividad de tres tipos de intervenciones de carácter poblacional.

2.- Conocer la cobertura y características (lugar y periodicidad) del cribado de cáncer de cuello uterino en la población de estudio.

3.- Estudiar el perfil de las mujeres que se incorporan al cribado de cáncer de cuello uterino y de las que no lo hacen.

Ajustarse al espacio disponible

Investigador/a principal: AMELIA ACERA PEREZ

Metodología (diseño, sujetos de estudio, variables, recogida y análisis de datos y limitaciones del estudio). *(Máximo 3 páginas)*

Se realizará un estudio coste-efectividad de tres estratégias de cribado poblacionales de cáncer cérvico-uterino. Se compararán estas estratégias con la estratégia de cribado oportunista actual mediante los datos del ensayo multicéntrico aleatório CRICERVA. El análisis se llevará a cabo desde una perspectiva del Sistema Nacional de Salud. Se tendrá en cuenta la preferencia del tiempo descontando los costes futuros y los resultados en salud al 3% (1)

DISEÑO: ensayo clínico aleatorizado pragmático de cuatro ramas con asignación por grupos, controlado, con evaluador ciego y multicéntrico. Unidad de aleatorización: ABS.
ÁMBITO: SAP de Cerdanyola-Ripollet, provincia de Barcelona, formado por 4 municipios y 5 ABS que da cobertura sanitaria a una población mayor de 14 años de 120.293 habitantes de entorno urbano. Cómo existirán cuatro grupos de estudio y hay 5 ABS, sólo se incluirán en el estudio las 4 ABS con criterios socioeconomicos más homogeneos.
POBLACION: 20.994 mujeres de 30 a 70 años con criterios de cribado incorrecto* (datos obtenidos del SIAP) adscritas a las ABS participantes en el estudio. *Cribado incorrecto (2): 1.- Sin citología en los 3 años previos, 2.- De 40 a 65 años que no se han hecho una citología en los 5 años previos, 3.- De más de 65 años sin historia previa de citologías o que se han hecho la última citología antes de los 60 años. **Criterios de exclusión: mujeres histerectomizadas, con historia actual de lesiones premalignas (AGUS, ASCUS, LSIL, HSIL), carcinoma in situ y cáncer cervicouterino, VIH positivas u otras causas de inmunosupresión (debido a que estas mujeres siguen un protocolo específico), las que residen durante más de 6 meses fuera del ámbito de estudio y las adscritas a las ABS de estudio pero con médico asignado en una UBA de otra zona diferente a la del estudio.
TAMAÑO MUESTRAL. El cálculo del tamaño de la muestra se ha realizado en base a detectar una diferencia de efectividad respecto al grupo control (GNI). Se ha calculado multiplicando el tamaño de un diseño aleatorio simple por el efecto diseño o factor de inflación. Para el diseño aleatorio simple, aceptando un riesgo alfa de 0.05 y un riesgo beta de 0.20 en un contraste bilateral, se precisan 59 sujetos en el primer grupo y 59 en el segundo para detectar una diferencia igual o superior al 28,4% en la cobertura del cribado de cáncer de cuello de útero entre ambos. Se asume una proporción de cobertura del cribado del 41,6% en el grupo no intervención. Se ha estimado una tasa de pérdidas de seguimiento del 20%. El cálculo de la muestra se ha realizado con el programa informático Granmo 5.2 para Windows. Considerando según la revisión de la literatura (3-5) un coeficiente de correlación intraclase de 0.05, y un número medio de mujeres de 30 a 70 años con cribado incorrecto por ABS de 3.500, resulta un efecto diseño de 176 de modo que se necesitan 20.768 mujeres con cribado incorrecto.

ANÁLISIS DE DECISIÓN.
Se desarrollará un modelo de Markov para simular la historia natural del cáncer cervicouterino y medir el impacto de tres estrategias diferentes de cribado en la progresión de la enfermedad y el coste. Este mismo modelo de decisión analítico ha sido utilizado en varios estudios de cribado de cáncer de cuello uterino (6-14). Los parámetros de este modelo, variables clínicas y costes, que se incluirán están detallados en los siguientes apartados de variables de este estudio. Se utilizará un Modelo de Markov para el análisis de decisión (15-16). La población se distribuira a través de las probabilidades de transición, entre los diferentes estados de salud a lo largo del tiempo. Los ciclos temporales del modelo de Markov seran de seis meses de duracion durante los cuales las mujeres podran cambiar de estado de salud o permanecer en el mismo. Estas probabilidades dependen de la edad, del estado del virus del papiloma humano, y de la historia de la enfermedad. Los 11 estados de

salud que se considerarán serán: mujer sana, infección VPH, lesión intraepitelial de bajo grado (L-SIL), lesión epitelial de alto grado (H-SIL), cáncer invasor estadio FIGO EIa (FIGOEIa), cáncer invasor estadio FIGO EIb (FIGOEIb), cáncer invasor estadio FIGO EIIa (FIGOEIIa), cáncer invasor estadio FIGO EIIb (FIGOEIIb), cáncer invasor estadio FIGO EIII (FIGOEIII), cáncer invasor estadio FIGO EIV (FIGOEIV), y muerte. Estos estados de salud recomendados por los expertos clínicos de este estudio son consistentes con los detallados, de manera extensa, por la literatura económica (6-14).

MEDIDAS DE EFECTIVIDAD CLÍNICA
Las principales medidas de efectividad de esta evaluación serán el número total de citologías realizadas, infecciones de HPV detectadas, lesiones en diferentes grados detectadas, episodios de cáncers detectados y número de muertes evitadas. La eficacia de las intervenciones se obtendrán del ensayo clínico CRICERVA y de extrapolaciones de la literatura, ya que se hará un corte transversal a los tres años, únicamente, para obtener los datos.

MEDIDAS DE COSTES
Técnicas de microcoste serán utilizadas para estimar los costes sanitários de las diferentes intervenciones para el cribado de cáncer cérvico-uterino y el tratamiento de los diferentes estados de salud (17). Los costes se presentarán en € del 2012 (año que se prevé finalizar el estudio). Datos primarios del ensayo clínico CRICERVA serán utilizados en la medida de lo posible. Los costes comprenderán los costes de diagnóstico, los costes de las diferentes intervenciones y, los costes de tratamiento para los diferentes estados de salud. El coste de diagnóstico comprenderá el coste de la citología, del test de la determinación del HPV y de la visita de control anual por el médico de Atención Primária. El coste de las intervenciones comprenderá el coste de un administrativo/a a jornada completa, de las cartas personalizadas para el cribado, de los folletos informativos y de las llamadas telefónicas. El coste del tratamiento dependerá del estadio de salud en el que se encuentre la mujer, pero puede comprender costes de las visitas al ginecólogo, citología, determinación del HPV, visitas de control con el médico, colposcopia, biopsia, conizaciones y en estadios más avanzados de la enfermedad, costes de histerectomía radical, radioterapia, quimioterapia, etc.

HORIZONTE TEMPORAL
El horizonte temporal del analisis sera el periodo de vida de la mujer.

ANALISIS COSTE-EFECTIVIDAD
El análisis coste-efectividad se llevará a cabo comparando los costes y efectividad, medida en unidades naturales, de las diferentes alternativas y practica habitual. En la mayoría de casos los datos serán obtenidos del ensayo clínico CRICERVA. La comparación se llevará a cabo utilizando la Razón Coste-Efectividad Incremental (RCEI), definida como la razón de la diferencia en costes y la diferencia en efectividad. Los resultados se expresarán en términos de €/por unidad de efectividad.

ESTUDIO DE LA INCERTIDUMBRE
Para estudiar la incertidumbre de este análisis, se llevará a cabo un análisis de sensibilidad determinístico univariante y multivariante de los parámetros que se consideren necesarios por los investigadores.

PLAN DE ANALISIS
Todo el analisis estadistico se llevara a cabo con el programa SPSS v.18 y Microsoft Excel. La evaluacion economica se llevara a cabo según el principio de tratar. Se realizara un analisis descriptivo de cada uno de los grupos. Se presentaran los valores medios y desviaciones estandar de

los costes y los intervalos de confianza al 95% correspondientes. El resultado final se presentara en terminos de efectividad y coste de cada una de las alternativas comparadas.

ASPECTOS ÉTICOS: los investigadores se comprometen a respetar las normas de Buena Práctica Clínica vigentes así como los requisitos de la Declaración de Helsinki y las cláusulas de las condiciones éticas generales y particulares relacionadas con el derecho a la intimidad, anonimato y confidencialidad. En ningún momento se registrará el nombre, apellidos o cualquier otro tipo de datos que lleve a la identificación de la mujer, la identificación se realizará mediante códigos numéricos. En este tipo de estudios, por desarrollarse en la práctica clínica habitual, la autorización y el apoyo han de ser dados por los representantes y autoridades de las colectividades involucradas con los que ya se cuenta, de modo que no es necesario el consentimiento informado individualizado, pero el equipo investigador ha decidido aún así que las mujeres que acudan a la consulta para realizarse la citología lo firmen. El protocolo ha sido evaluado por el CEIC del IDIAP Jordi Gol.
LIMITACIONES:*Con la aleatorización por grupos evitaremos la posible contaminación que puede producirse entre las intervenciones al realizarse en un mismo emplazamiento.*Puesto que no se puede aplicar la técnica de enmascaramiento del doble ciego por las características del estudio, se aplicará la evaluación enmascarada de la respuesta para que la medición de las variables dependientes se realice e interprete de la misma forma para todos los grupos.*La posible pérdida de información que se podría producir en las mujeres que se realizan el cribado fuera de la sanidad pública si no se ponen en contacto con el equipo investigador se minimizará con la llamada telefónica de repesca que se realizará cuando la mujeres no acudan a la visita.*La dificultad de entendimiento con mujeres de otros países por la barrera idiomática se intentará resolver con los mediadores culturales de cada localidad.*Desde el 2007 en el ámbito de estudio el personal administrativo actualiza las direcciones postales de los usuarios que acuden al ABS, se cuenta con un registro postal bastante exacto, reduciéndose así las posibles pérdidas de las cartas. En caso de que se produzca esta situación se resolverá como se especifica en la fase 8 del estudio.

Anexo 2: referencias bibliográficas de la Metodología.

MEMORIA DE SOLICITUD DEL PROYECTO DE INVESTIGACIÓN

Expediente nº

PI10/01275

Investigador/a principal: AMELIA ACERA PEREZ

Plan de trabajo (etapas de desarrollo y distribución de tareas de todo el equipo investigador, y las asignaciones previstas para el personal técnico que se solicita. Indicar también el lugar de realización del proyecto). *(Máximo 1 página)*

La secuencia de actividades sigue el esquema: Actividad (A)-Descripción de la actividad-Mes de realización (M)-Responsable de la acción-Lugar de realización. Las abreviaturas se especifican en el anexo 1.

FASE 1. Difusión del proyecto: A 1: Difusión del proyecto por todas las ABS del ámbito de estudio (M1). A. Acera, JM. Bonet, P. Soteras, P. Hidalgo. ABS del ámbito de estudio. A 2: Elaboración del protocolo definitivo (M1-2). EI. CAP Fontetes.

FASE 2. Selección de la población de estudio: A3: Obtención de los listados de mujeres que cumplen los criterios que definen la población de estudio (M1-2). N. Sanchez. CAP II Cerdanyola-Ripollet.

FASE 3. Creación de los grupos de estudio: A4: Aleatorización de las intervenciones (M3). JM. Bonet y M. Trapero. CAP Fontetes.

FASE 4. Prueba piloto: A5: Desarrollo de la prueba piloto (M3-4). EI y personal técnico que se solicita. ABS del ámbito de estudio. A6: Evaluación de la prueba piloto e incorporación de las modificaciones oportunas para el buen desarrollo del estudio (M4). EI y personal técnico que se solicita. CAP II Cerdanyola-Ripollet.

FASE 5. Elaboración del material de estudio y formación de los profesionales sanitarios: A7: Elaboración de todo el material necesario para el desarrollo del estudio (cartas, dípticos, CRDe, etc), consensuar circuitos, fijar secuencia de actividades, identificar y resolver problemas logísticos (M5-6). EI y personal técnico que se solicita. CAP II Cerdanyola-Ripollet. A8: Formación de todos los profesionales participantes (ginecólogos y matronas) en el manejo del CRDe y de la explicación exhaustiva de las fases del estudio (M6). A. Acera, P. Soteras, A. Rodriguez, P. Hidalgo. CAP II Cerdanyola-Ripollet.

FASE 6. Intervención (sólo GI): A9: Implantación, de carácter secuencial, de Ias tres intervenciones (M7-27). EI y personal técnico que se solicita. ABS del ámbito de estudio.

FASE 7. Inclusión de las mujeres en el cribado: A10: Recogida de datos en el CRDe de las mujeres que se incorporen al programa de cribado del GNI (M7-27). Ginecólogos y matronas. ABS del ámbito de estudio. A11: Recogida de datos en el CRDe de las mujeres que se incorporen al programa de cribado de los G1, 2 y 3 (M7-27). Ginecólogos y matronas. ABS del ámbito de estudio.

FASE 8. Nuevo aviso a las mujeres que no acudan a la cita establecida (sólo GI): A12: Llamada telefónica a las mujeres de los GI que no acudan a la cita establecida (M7-29). Ginecólogos, matronas y personal técnico que se solicita. ABS del ámbito de estudio.

FASE 9. Cierre de la recogida de datos: A13: Controles de calidad de los datos registrados en el CRDe (M7-30). N. Sanchez. CAP II Cerdanyola-Ripollet. A14: Evaluación de las variables dependientes a los 3 meses de la intervención en las mujeres del GI y 3 meses antes del cierre de la base de datos en las mujeres del GNI (M10-30). EI. CAP II Cerdanyola-Ripollet. A15: Cierre de la base de datos (M30). JM Bonet y M. Trapero. CAP II Cerdanyola-Ripollet.

FASE 10. Análisis de los datos y difusión de los resultados: A16: Memoria primera anualidad (M12). EI. CAP II Cerdanyola-Ripollet. A17: Memoria segunda anualidad (M24). EI. CAP II Cerdanyola-Ripollet. A18: Análisis final de los datos del estudio (M30-35). A. Acera, JM Bonet, M. Trapero, A. Rodriguez. CAP II Cerdanyola-Ripollet. A19: Elaboración del informe final (M36). EI. CAP II Cerdanyola-Ripollet. A20: Difusión de los resultados en diferentes escenarios científicos (a partir del M24). EI.

Actividades que se desarrollarán a lo largo de todo el estudio: A21: Revisión bibliográfica (M1-M36). EI. CAP II Cerdanyola-Ripollet. A22: Coordinación del estudio y resolución de dudas (M1-M36). A. Acera, P. Soteras, A. Rodríguez, P. Hidalgo. CAP II Cerdanyola-Ripollet.

MEMORIA DE SOLICITUD DEL PROYECTO DE INVESTIGACIÓN

Expediente nº

PI10/01275

Investigador/a principal: AMELIA ACERA PEREZ

Experiencia del equipo investigador sobre el tema. *(Máximo 1 página)*

Se trata de un equipo multidisciplinar formado por ginecólogos, matronas, médicos de familia, técnico de salud e informático. Se diferencian dos perfiles: personal asistencial con gran experiencia en la práctica clínica y la actividad preventiva en el ámbito de la AP y personal técnico con gran destreza en metodología de la investigación y el manejo de bases de datos, necesario para el buen desarrollo de un proyecto de investigación. A pesar de la reciente formación del equipo investigador, el grupo tiene intereses comunes en desarrollar una línea de investigación en el área de Salud Sexual y Reproductiva de la mujer, así como una proyección de futuro y perdurabilidad.
Algunos de los miembros del equipo tienen una sólida formación en el ámbito de la salud sexual y reproductiva de la mujer, no sólo por llevar muchos años desarrollando su actividad asistencial en este campo sino por su participación y colaboración en diversas actividades científicas tales como:
- A. Acera fue miembro en 1993 del grupo de trabajo para la implantación del cribado poblacional del cáncer de mama en el área del Vallés Occidental. - A. Acera es investigadora colaboradora del estudio "Cribado pre-natal del Síndrome de Down mediante test de contingencia en el primer trimestre, estudio multicéntrico de aplicación del test combinado seguido del estudio selectivo de marcadores ecográficos secundarios" (PI060151), Proyecto de Investigación en Salud 2006, Instituto de Salud Carlos III.
- A. Acera y P. Soteras han sido colaboradoras en el estudio "Prevención del cáncer cervico-uterino y detección del virus del papiloma humano", realizada por el Instituto Catalán de Oncología, el Instituto Catalán de la Salud y la Región Sanitaria Centro, llevado a cabo durante el año 2004.
- P. Soteras es investigadora colaboradora del estudio "Estado nutricional de yodo en la población gestante de Cataluña: estudio de hábitos higiénico-dietéticos y yodurias" (PI071265), Proyecto de Investigación en Salud 2007, Instituto de Salud Carlos III. - P. Soteras es coautora de la Guía de Práctica Clínica sobre Contracepción de Emergencia elaborada en el 2004 por el Departamento de Salud de Cataluña.
- A. Rodríguez realizó el proyecto de investigación en Enfermería Obstétrico-Ginecológica sobre patología cervicouterina: "Análisis de la actividad para el cribado del cáncer de cuello uterino en el SAP de Cerdanyola-Ripollet desde el 2003 al 2007".
Otros miembros del equipo tienen una novel pero creciente trayectoria en investigación, siendo miembros de diferentes equipos de proyectos que han conseguido financiación en convocatorias competitivas:
- JM. Bonet, ha participado en diferentes proyectos como investigador colaborador y actualmente es investigador principal de un estudio de intervención sobre cuidadores de personas dependientes que ha sido doblemente premiado en el 2008 por la SCMFyC y la Región Sanitaria de Barcelona.
- P. Hidalgo, es miembro de diferentes grupos de trabajo del SAP Cerdanyola-Ripollet y nacional (ecografía de SEMERGEN), habiendo elaborado diferentes protocolos y guías.
- M. Trapero-Bertran, ha participado como investigadora colaboradora en diferentes proyectos de economía de la salud y actualmente está cursando su doctorado, sobre la incorporación de los efectos externos del tabaco en las evaluaciones económicas, en el Health Economics Research Group (HERG), universidad de Brunel, Reino Unido. Es profesora asociada e investigadora en la UPF. También es secretaria de la Asociación de Economía de la Salud (AES).
- N. Sánchez, ha participado como autor en algunas comunicaciones en el ámbito de AP y tiene una gran experiencia en la creación de bases de datos con normas de coherencia interna así como en su manejo y explotación.
- JM Manresa ha participado en diferentes proyectos FIS (953203358-FIS-02 y 953202361-FIS-09) como investigador colaborador y actualmente en el proyecto FIS PI080956/2008. En este proyecto proporciona soporte metodológico y estadístico.

MEMORIA DE SOLICITUD DEL PROYECTO DE INVESTIGACIÓN

Expediente nº

PI10/01275

Investigador/a principal: AMELIA ACERA PEREZ

Plan de difusión:
1. Relevancia del proyecto en cuanto a su impacto clínico, asistencial y/o desarrollo tecnológico.
2. Relevancia del proyecto en cuanto a su impacto bibliométrico.

1.- Planteamos aprovechar los recursos disponibles en el sistema público de salud con una reorganización de la carga de trabajo, un aprovechamiento de los sistemas informáticos y de la cercanía de los centros de AP para aumentar la cobertura y disminuir el cáncer de cuello uterino mejorando la salud de las mujeres a corto y largo plazo. En el marco en que la disponibilidad de intervenciones sanitarias supera ampliamente la capacidad de la sociedad para pagarlas, la evaluación económica, podrá valorar la conveniencia de cada una de las tres intervenciones planteadas. Los resultados que se obtengan serán generalizables a otros territorios porque no se tratan de intervenciones demasiado específicas y serán un reflejo bastante exacto de la realidad del cribado desde la AP. El proyecto propuesto se enmarca en una de las líneas de la estrategia en cáncer del SNS, concretamente en el objetivo 7 de la Detección Precoz, (se desarrollarán programas de seguimiento organizado de aquellas personas con riesgo elevado de padecer cáncer de mama, colorrectal y de cérvix). También se puede clasificar en la línea 3 (Fomento de la investigación en Salud Pública, Salud Ambiental y Salud Laboral, y Dependencia y Servicios de Salud, para la mejor calidad de vida funcional de la población española) de investigación prioritaria de la modalidad B de la presente convocatoria. 2.- Se publicará el protocolo del estudio en el BioMed Central Health Services Research (factor de impacto: 1.36) o Public Health (factor de impacto: 1.63). Los resultados generados serán motivo de publicación en revistas científicas nacionales e internacionales de contenido tanto médico-clínico, de Salud Pública, como de Economía de la Salud, dado el gran interés que supone demostrar empíricamente el beneficio que un programa de cribado poblacional bien diseñado puede reportar en la salud de las mujeres desde el punto de vista de la eficiencia.

Medios disponibles para la realización del proyecto.

Nuestro Servicio de Atención Primaria (SAP) cuenta con dos Áreas Básicas de Salud (ABS) en Cerdanyola, una en Ripollet, una en Barberá del Vallés y una en Badía del Vallés. Todos los centros que participan en el estudio están equipados con los medios necesarios para asegurar la consecución del estudio propuesto.

Todas las ABS disponen de varias consultas para realizar la anamnesis y exploración física de las mujeres que se incluirán en el cribado, de personal altamente entrenado (matronas) para la toma de la muestra citológica y de nuevo material informático, suministrado recientemente por el SAP, para el registro de los datos, actividad fundamental en el desarrollo de cualquier estudio. En definitiva, todos los investigadores que colaborarán, trabajan en la red de Atención Primaria del Instituto Catalán de la Salud (ICS) y podrán disponer de todos los medios estructurales y personales (soporte informático y metodológico) del ICS y del IDIAP Jordi Gol.

Ajustarse al espacio disponible

MEMORIA DE SOLICITUD DEL PROYECTO DE INVESTIGACIÓN

Expediente nº

PI10/01275

Investigador/a principal: AMELIA ACERA PEREZ

Justificación detallada de la ayuda solicitada. *(Máximo 1 página)*

Gastos de ejecución:

A).- Adquisición de bienes y contratación de servicios:

CONTRATACIÓN DE SERVICIOS DE SOPORTE ADMINISTRATIVO: se trata de un estudio multicéntrico que requiere de una buena monitorización y seguimiento, incluyendo visitas a los diferentes centros de salud, llamadas telefónicas y una continua supervisión. Dado que los miembros del equipo investigador realizan asistencia a jornada completa se considera fundamental para el buen desarrollo del proyecto la contratación de servicios a personal administrativo. Este personal deberá tener conocimientos en el área sanitaria. Las actividades que desarrollaría en el estudio serían entre otras: personalización de las cartas, envío del material necesario para cada tipo de intervención, llamadas telefónicas, creación de un Cuaderno de Recogida de Datos Electrónico y registro en el mismo de los casos que no acudan a la consulta para incorporarse al cribado.

GASTOS GENERADOS POR LA INTERVENCIÓN:

- Material de oficina, didáctico, gastos de correspondencia y de administración: son importantes en cualquier proyecto de investigación pero en este estudio resultan imprescindibles, pues se basa en la distribución de cartas de invitación para participar en el cribado así como folletos informativos y llamadas telefónicas. Contemplamos la compra de papel, sobres, gastos generados por los envíos postales. Los folletos informativos que se enviarán a uno de los grupos de estudio serán proporcionados gratuitamente por el Departament de Salut.
- Llamadas telefónicas: se contratará un paquete de llamadas por móvil a través de la CTTI
- Material informático: para la buena coordinación, emisión de etiquetas, cartas personalizadas y del desarrollo del proyecto consideramos necesario un ordenador portátil con suficiente capacidad en la gestión de las bases de datos.
- Gastos en las pruebas de Hibrid Capture para el HPV generadas expresamente por la intervención en grupos de edad sin cribado habitual y no son asumibles por el Sistema Público de Salud debido a que las mujeres mayores de 65 años no entran dentro del protocolo establecido.

GASTOS DE PUBLICACIÓN Y PETICIÓN DE ARTÍCULOS: está prevista la contratación de traductores profesionales para la publicación del protocolo y de los artículos que se generen a partir del estudio en revistas internacionales con factor impacto relevante. El equipo investigador considera relevante conocer todo lo que se está publicando en la literatura científica nacional e internacional en el campo de la patología y cribado de cáncer de cuello uterino.

B).- Viajes y dietas:

Durante la fase inicial el equipo investigador deberá desplazarse a las 5 ABS del ámbito de estudio, distantes entre si entre 7 y 10 km., para difundir y explicar el proyecto,

Se ha calculado el gasto generado por este motivo en base a un desplazamiento promedio según la dispersión geográfica de las ABS participantes. Se contemplan los gastos de desplazamiento generados por las reuniones mensuales de coordinación del equipo así como de monitorización del estudio y de resolución de las posibles eventualidades que surjan. También se considera una partida destinada a los gastos generados por asistencia a eventos científicos para la difusión de los resultados obtenidos, una inscripción a un congreso nacional y otra inscripción a un congreso internacional.

MEMORIA DE SOLICITUD DEL PROYECTO DE INVESTIGACIÓN

Expediente nº

PI10/01275

Investigador/a principal: AMELIA ACERA PEREZ

PRESUPUESTO SOLICITADO	
**1. Gastos de personal**	**Euros**
**SUBTOTAL**	
**2. Gastos de ejecución**	
**a) Adquisición de bienes y contratación de servicios** (inventariable, fungible y otros gastos).<br>CONTRATACIÓN DE SERVICIO DE SOPORTE ADMINISTRATIVO:<br>GASTOS GENERADOS POR LA INTERVENCIÓN:<br>- Cartas (28.300 €) + Material de oficina (3000 €)<br>- Llamadas telefónicas<br>- 1 ordenador portátil: 1.600 €<br>GASTOS DE PUBLICACIÓN:<br>- Del protocolo en revista electrónica con factor de impacto (BioMed Central) (traducción al inglés y pago para su publicación) (1.800 €)<br>- De artículos en revistas con factor de impacto (traducción) (1.200 €)<br>PRUEBAS DE HIBRIDD CAPTURE (2.200 determinaciones)	31.680,00 €<br><br>31.300,00 €<br>2.250,00 €<br>1.600,00 €<br>3.000,00€<br><br><br>48.400,00 €
**SUBTOTAL**	118.230,00 €
**b) Viajes y dietas**<br>Desplazamientos del equipo investigador a las ABS para difusión del proyect[illegible] monitorizar y coordinar el estudio<br>Inscripción, alojamiento, viajes y dietas a un congreso nacional para una persona<br>Inscripción, alojamiento, viajes y dietas a un congreso internacional para una persona	500,00 €<br><br>1.000,00 €<br><br>2.000,00 €
**SUBTOTAL**	3.500,00 €
**SUBTOTAL GASTOS EJECUCIÓN**	121.730,00 €
**TOTAL AYUDA SOLICITADA**	121.730,00 €

MEMORIA DE SOLICITUD DEL PROYECTO DE INVESTIGACIÓN

Expediente nº

PI10/01275

Investigador/a principal: AMELIA ACERA PEREZ

ANEXOS

ANEXOS

Anexo 1. Abreviaturas
Anexo 2. Referencias bibliográficas de la metodología.
Anexo 3. Cuaderno de recogida de datos.
Anexo 4. Evaluación Económica
Anexo 5. Algoritmo del estudio.
Anexo 6. Carta de invitación al cribado
Anexo 7. Plan de trabajo para el cribado de mujeres sanas del protocolo de las actividades para el cribado de cáncer de cuello uterino en Atención Primaria.
Anexo 8 Hoja de información a la paciente.
Anexo 9 Consentimiento informado

Anexo 1.- ABREVIATURAS

AATM	Agència d'Avaluació de Tecnologia i Recerca Mèdiques
ABS	Área Básica de Salud
AGUS	Células glandulares atípicas de significado indeterminado (atypical glandular cells of undetermined significance)
ASCUS	Células escamosas atípicas de significado indeterminado (atypical squamous cells of undetermined significance)
CAP	Centro de Atención Primaria
CEIC	Comité de ética de investigación clínica
CRDe	Cuaderno de recogida de datos electrónico
EAP	Equipo de Atención Primaria
e-CAP	Historia clínica informatizada
EI	Equipo investigador
GI	Grupo intervención
GNI	Grupo de no intervención
HC2	Hybrid Capture 2
HSIL	Lesión intraepitelial escamosa de alto grado (high grade squamous intraepitelial lesion)
ICO	Instituto Catalán de Oncología
ICS	Instituto Catalán de la Salud
IDIAP	Instituto de investigación en Atención Primaria
LSIL	Lesión intraepitelial escamosa de bajo grado (low grade squamous intraepitelial lesion)
PASSIR	Programa de Atención a la Salud Sexual y Reproductiva
SAP	Servicio de Atención Primaria
USR	Unidad de soporte a la investigación
VHS	Virus del Herpes Simple
VIH	Virus de la inmunodeficiencia humana (human immunodeficiency virus)
VPH	Virus del papiloma humano (human papilloma virus)

Anexo 2. REFERENCIAS BIBLIOGRÁFICAS DE LA METODOLOGÍA

*1.- Lopez Bastida J, Oliva J, Antonanzas F, Garcia-Altes A, Gisbert R, Mar J, Puig-Junoy J. Propuesta de guía para la evaluación económica aplicada a las tecnologías sanitarias. Gaceta Sanitaria 2009. doi:10.1016/j.gaceta.2009.07.011.*2.-Protocolo de las actividades para el cribado del cáncer de cuello uterino en Atención Primaria [monografía en Internet]. Barcelona: Generalitat de Catalunya, Departamento de Salud; 2006 [citado el 15 de noviembre de 2008]. Disponible en: http://www.gencat.cat/salut/depsalut/pdf/collut.pdf; *3.- A 2.- Adams G, Gulliford MC, Ukoumunne OC, Eldridge S, Chinn S, Campbell MJ. Patterns of intra-cluster correlation from primary care research to inform study design and analysis. J Clin Epidemiol. 2004; 57:785-794.*4. Parker DR, Evangelou E, Eaton CB. Intraclass correlation coefficients for cluster randomized trials in primary care: the cholesterol education and research trial (CEART). Contemp Clin Trials. 2005; 26:260-267. * 5.- Ukoumunne OC, Gulliford MC, Chinn S, Sterne JA, Burney PG. Methods for evaluating area-wide and organisation-based interventions in health and health care: a systematic review. Health Technol Assess. 1999; 3(5):iii-92. *6-. Goldie S J, Gaffikin L, Goldhaber-Fiebert J D, Gordillo-Tobar A, Levin C, Mahé C, et al. Cost-effectiveness of Cervical-Cancer Screening in Five Developing Countries. The New England Journal of Medicine. 2005; 353:2158-68. *7-. Siebert U, Sroczynski G, Hillemanns P, Engel J, Stabenow R, Stegmaier C, et al. The German Cervical Cancer Screening Model: development and validation of a decision-analytic model for cervical cancer screening in Germany. European Journal of Public Health. 2006; 16(2):185-92. *8-. Mandelblatt J S, Lawrence W F, Mizell Womack S, Jacobson D, Yi B, Hwang Y, et al. Benefits and Costs of Using HPV Testing to Screen for Cervical Cancer. JAMA. 2002; 287(18):2372-81. *9-. Goldie S, Kuhn L, Denny L, Pollack A, Wright T C. Policy Analysis of Cervical Cancer Screening Strategies in Low-Resource Settings. JAMA. 2001; 285(24):3107-15. *10-. Berkhof J, de Bruijne M C, Zielinski G D, Bulkmans N W J, Rozendaal L, Snijders P J F, et al. Evaluation of cervical screening strategies with adjunct high-risk human papillomavirus testing for women with borderline or mild dyskaryosis. International Journal of cancer. 2006; 118:1759-68. *11-. Mittendorf T, Petry K U, Iftner T, Greiner W, von der Schulenburg J M. Economic evaluation of human papillomavirus screening in Germany. European Journal of Health Economics. 2003; 4(3):209-15. *12-. Vijayaraghavan A, Efrusy M, Lindeque G, Dreyer G, Santas C. Cost effectiveness of high-risk HPV DNA testing for cervical cancer screening in South Africa. Gynecologic Oncology. 2009; 112(2):377-83. *13-. Kim J J, Wright T C, Goldie S J. Cost-effectiveness of Alternative Triage Strategies for Atypical Squamous Cells of Undetermined Significance. JAMA. 2002; 287(18):2382-90. *14-. Holmes J, Hemmett L, Garfield S. The cost-effectiveness of human papillomavirus screening for cervical cancer. A review of recent modeling studies. European Journal of Health Economics. 2005; 6(1):30-7. *15.- Briggs A, Sculpher M. An introduction to Markov modelling for economic evaluation. Pharmacoeconomics, 1998; 13(4):397-409. *16.- Karnon J, Brown J. Selecting a decision model for economic evaluation: a case study and review. Health Care Management Science, 1998; 1:133-140. *17.-Drummond MF, Sculpher MJ, Torrance GW, O'Brien BJ, Stoddart GL. Methods for the economic evaluation of health care programmes. Oxford university Press, 2005. 3rd edition.

Anexo 3. CUADERNO DE RECOGIDA

Dades de l'usuari (com a mínim el CIP i data de visita)

Plantilla del CRD de l'estudi CRICERVA

1. No presentat en 1ª visita

2. Nacionalitat:

3. Antecedents patolgics
- Cap antecedent
- Histerectomia
- Càncer uterí
- Càncer de coll uterí
- Altres
- No contesta

4. S'ha fet mai una citologia, amb quina periodicitat?
- Mai
- Cada any
- Cada 2 anys
- Cada 3 anys
- Cada 4 anys
- Cada 5 anys o mes
- No contesta

5. Si fa mes de 3 anys que no s'ha realitzat cap citologia, quin ha estat el motiu?
- No m'agrada
- Tinc por
- No creia que era important
- No tenia informació que això fos necesari

6. Lloc on li practiquen el cribatge
- Sistema públic
- Privat
- Mixte (privat i públic)
- Cap
- No contesta

7. Determinació del VPH prèvia
- Si Any:
- No
- No contesta

8. Viu amb parella?
- Si
- No
- No contesta

9. Fills
- Nombre:

10. Cuidador
- Nombre:

11. Màxim nivell d'estudis cursats
- Analfabet, sense estudis.
- Primaris incomplerts
- Primaris (EGB o similar)
- Secundaris (FP, BUP, COU o similar)
- Superiors (Universitaris)
- No contesta

12. Situació laboral actual
- Actiu
- Feines de la llar
- Estudiant
- Baixa laboral
- Invalidesa Transitòria
- Jubilat
- No contesta

13. Ingressos mensuals de la dona
- de 0 a 600€
- de 601 a 1000€
- de 1001 a 2000€
- mes de 2000
- No contesta

14. Ingressos mensuals del nucli familiar
- de 0 a 600
- de 601 a 1000
- de 1001 a 2000
- mes de 2000
- No contesta

Les dades: Data de naixament i direcció s'obtenen de la base de dades del SIAP

Anexo 4. Ensayo clínico CRICERVA

DISEÑO: ensayo clínico aleatorizado pragmático de cuatro ramas con asignación por grupos, controlado, con evaluador ciego y multicéntrico. Unidad de aleatorización: ABS.
ÁMBITO: SAP de Cerdanyola-Ripollet, provincia de Barcelona, formado por 4 municipios y 5 ABS que da cobertura sanitaria a una población mayor de 14 años de 120.293 habitantes de entorno urbano. Cómo existirán cuatro grupos de estudio y hay 5 ABS, sólo se incluirán en el estudio las 4 ABS con criterios socioeconomicos más homogeneos.
SUJETOS DE ESTUDIO: 20.994 mujeres de 30 a 70 años con criterios de cribado incorrecto* (datos obtenidos del SIAP) adscritas a las ABS participantes en el estudio. *Cribado incorrecto (1): 1.- Sin citología en los 3 años previos, 2.- De 40 a 65 años que no se han hecho una citología en los 5 años previos, 3.- De más de 65 años sin historia previa de citologías o que se han hecho la última citología antes de los 60 años. **Criterios de exclusión: mujeres histerectomizadas, con historia actual de lesiones premalignas (AGUS, ASCUS, LSIL, HSIL), carcinoma in situ y cáncer cervicouterino, VIH positivas u otras causas de inmunosupresión (debido a que estas mujeres siguen un protocolo específico), las que residen durante más de 6 meses fuera del ámbito de estudio y las adscritas a las ABS de estudio pero con médico asignado en una UBA de otra zona diferente a la del estudio. **Tamaño muestral: el cálculo del tamaño de la muestra se ha calculado multiplicando el tamaño de un diseño aleatorio simple por el efecto diseño o factor de inflación. Para el diseño aleatorio simple, aceptando un riesgo alfa de 0.05 y un riesgo beta de 0.20 en un contraste bilateral, se precisan 59 sujetos en el primer grupo y 59 en el segundo para detectar una diferencia igual o superior al 28,4% en la cobertura del cribado de cáncer de cuello de útero entre ambos. Se asume una proporción de cobertura del cribado del 41,6% en el grupo no intervención. Se ha estimado una tasa de pérdidas de seguimiento del 20%. El cálculo de la muestra se ha realizado con el programa informático Granmo 5.2 para Windows. Considerando según la revisión de la literatura (2-4) un coeficiente de correlación intraclase de 0.05, y un número medio de mujeres de 30 a 70 años con cribado incorrecto por ABS de 3.500, resulta un efecto diseño de 176 de modo que se necesitan 20.768 mujeres con cribado incorrecto.
VARIABLES: las categorías de las variables se especifican en el CRD (anexo 3). Fuente de información: historia clínica informatizada (e-CAP) y entrevista individualizada en la consulta o telefónica: =Dependientes: *Objetivo Principal: Participación en el cribado (si, no): mujer que acude a la cita establecida en la carta de invitación para realizarse la citología sin necesidad de la llamada de repesca. *Objetivo específico 3: nº de infecciones de VPH, de lesiones epiteliales, de cáncer de cuello uterino y de muertes evitadas por cada una de las intervenciones. Las variables del modelo económico se especifican en el anexo 4. =Independientes: **Variables del sujeto: - Principal: grupo de estudio (1, 2, 3 y no intervención). - Otras: *Administrativas: ABS, lugar de residencia; *Sociodemográficas: fecha nacimiento, país de origen, nacionalidad española, máximo nivel de estudios alcanzado, situación laboral, ingresos mensuales de la mujer, ingresos mensuales del núcleo familiar, clase social, estado civil, tiene hijos, cuidador de personas dependientes; *Relacionadas con antecedentes de patología cervicouterina; *Relacionadas con el cribado: lugar en el que se hace el cribado y periodicidad con la que se hace el cribado, resultado de la última citología, determinación del VPH previa; *Relacionadas con la intervención: acude a la primera cita, motivo de no acudir a la primera cita, acude a la segunda cita. **Variables del ABS (información obtenida mediante encuesta al ASSIR Metropolitana Nord): antigüedad ABS, nº centros, población asignada, población atendida, edad media población asignada, centro docente de residentes, nº ginecólogos, nº matronas, nº horas personal ASSIR/semana.
DESARROLLO DEL ESTUDIO: en el anexo 5 se detallan las fases con un algoritmo de decisión. *FASE 1. Difusión del proyecto: en las ABS participantes para informar a los profesionales sanitarios del estudio que se llevará a cabo. *FASE 2. Selección de la población de estudio: a través del SIAP del SAP se obtendrán los listados de mujeres que cumplen los criterios que definen la

población de estudio. *FASE 3. Creación de los grupos de estudio: la unidad de aleatorización será el ABS y mediante aleatorización simple con números aleatorios generados con ordenador, los municipios se distribuirán a 4 grupos de estudio: GNI, grupo intervención (GI) 1, GI2 y GI3. *FASE 4. Prueba piloto: se realizará en un grupo de mujeres (25-30) que serán excluidas del estudio para que no afecten a los resultados finales. Se pretende evaluar el material informativo que se utilizará en el estudio, el proceso de recogida de datos mediante el CRDe creado para este estudio con reglas de coherencia interna para garantizar un control de calidad de los datos, el funcionamiento de los circuitos establecidos y las dificultades encontradas en los diferentes tipos de intervención. Una vez realizada esta evaluación se establecerán e incorporarán las modificaciones oportunas para el mejor desarrollo del estudio. *FASE 5. Elaboración del material de estudio y formación de los profesionales sanitarios: se elaborará el material necesario para el desarrollo del estudio (cartas, CRDe, etc), se consensuarán los circuitos, se identificarán y resolverán los problemas logísticos. Se formará a todos los profesionales participantes (ginecólogos y matronas) en el manejo del CRDe y se explicarán exhaustivamente las fases del estudio. *FASE 6. Intervención: en el GNI no se hará ninguna intervención, se seguirá la práctica clínica habitual del área sanitaria de estudio, que consiste en realizar una citología a la mujer que acude de forma oportunista a la consulta y no tiene citología en los 3 años previos, incluyendola posteriormente en un sistema de cita programada a 3 años, sistema mediante el cual reciben una carta con la cita de su próxima citología un mes y medio antes de la misma (estrategia de programación específica de este área sanitaria). En los otros 3 grupos se practicarán 3 tipos de intervenciones diferentes: *GI1: se enviará una carta de invitación personalizada para la participación en el cribado firmada por su médico de AP y profesionales del ASSIR. En ella se informará sobre el cáncer de cuello uterino y su cribado, se incluirá una fecha de citación, que la citología será realizada por una mujer y un teléfono de consulta en caso de dudas o de cambio de cita. Se especificará que si realiza el cribado fuera del sistema sanitario público, se ponga en contacto con el equipo investigador para registrar las variables de estudio (anexo 6). *GI2: se enviará la misma carta de invitación que en el GI1 y además un folleto informativo sobre el cribado de cáncer de cuello uterino vigente en Cataluña y editado por el Departamento de Salud (anexo 7). De este modo se valorará la repercusión que tiene la participación informada. *GI3: se hará la misma intervención que en el GI2 pero además, se realizará una llamada telefónica 3 días antes de la cita fijada en la carta de invitación como recordatorio de la visita. De este modo se valorará la repercusión que tiene la llamada telefónica recordatoria. Estas intervenciones serán secuenciales en el tiempo, se harán periódicamente hasta llegar a toda la población de estudio, empezando por las mujeres de mayor edad (70 años). *FASE 7: Inclusión de las mujeres en el cribado (tanto en el GNI como en los GI1, 2 y 3): las mujeres que acudan al programa de cribado, sea espontáneamente en las mujeres del GNI o por acudir a la cita establecida en las mujeres de los GI, se incorporarán al circuito del cribado de según las pautas establecidas en el protocolo vigente en Cataluña (anexo 8) y teniendo en cuenta el sistema de programación instaurado en el ámbito de estudio (ver fase 5). Cuando acudan a la consulta se les informará en que consiste el estudio verbalmente y por escrito (anexo 9) y se les pedirá que firmen el consentimiento informado (anexo 10). En el GNI se recogerán todas las variables excepto las relacionadas con la intervención. En ningún momento se registrará el nombre, apellidos o cualquier otro tipo de dato que lleve a la identificación de la mujer, se asignarán códigos numéricos. *FASE 8: Nuevo aviso a las mujeres que no acudan a la cita establecida (sólo en los GI): aquellas mujeres, independientemente del grupo de intervención al que pertenezcan, que no acudan a la cita establecida recibirán una llamada 10 días después, en la cual se les preguntará el motivo de no acudir a la cita. Si el motivo es no haber recibido la carta, se comprobará la dirección postal y si es diferente a la que consta en el e-CAP, se actualizará para un nuevo envío de la carta. Si están interesadas en incorporarse al cribado se les proporcionará una nueva cita, y si no lo están se les preguntará por las variables especificadas en el CRDe. Sólo se considerarán éxitos de participación en el cribado (variable dependiente) aquellas mujeres que acuden a la primera cita establecida en la

carta para realizarse la citología sin necesidad de este nuevo aviso telefónico (llamada de repesca). *FASE 9: Cierre de la recogida de datos: 6 meses antes de la finalización del estudio cesarán las intervenciones y la recogida de datos. Gracias a los controles periódicos de los datos registrados en el CRDe se habrán localizado y subsanado las incoherencias surgidas teniendo así una base de datos depurada y de calidad. *Fase 10: Análisis de los datos y difusión de los resultados: se valorará la efectividad de las intervenciones propuestas en términos de diferencia de la cobertura del cribado y de coste-efectividad entre los grupos de intervención y no intervención. El análisis de los datos estará cegado, no se conocerá a que grupo de estudio pertenece cada mujer. Se elaborará el informe final y se hará difusión de los resultados en diferentes escenarios científicos. ANÁLISIS ESTADÍSTICO: los datos serán analizados de acuerdo con la guía del Consort Cluster (5) y las comparaciones entre grupos se basarán en el principio de intención de tratar. Se realizará análisis de la comparabilidad basal de los grupos de estudio en relación con las variables estudiadas y estadística descriptiva de todas las variables recogidas. Se utilizará el test de t-Student o la U-Mann-Whitney (según la distribución de frecuencias) para la comparación de medias entre dos categorías, ANOVA o Wilcoxon (según la distribución de frecuencias) para la comparación de medias entre más de dos categorías, Ji-cuadrado o Ji-cuadrado de tendencia para la comparación de variables categóricas. Se efectuará un análisis con un modelo de regresión logística, ajustando los errores estándard por el efecto cluster teniendo en cuenta que cada ABS recibe un tipo de intervención diferente, para evaluar la asociación entre cada una de las variables dependientes descritas y las variables independientes que hayan resultado ser de interés en el análisis bivariado. Todos los análisis se harán ajustando por los potenciales factores de confusión y las posibles variables clínicas relevantes. Todas las pruebas se realizarán con un nivel de confianza del 95% a nivel bilateral y los paquetes estadísticos serán: SPSS16, STATA10 y HLM6. El modelo de evaluación económica se explica en el anexo 4.

.

Anexo 5. Algoritmo del estudio.

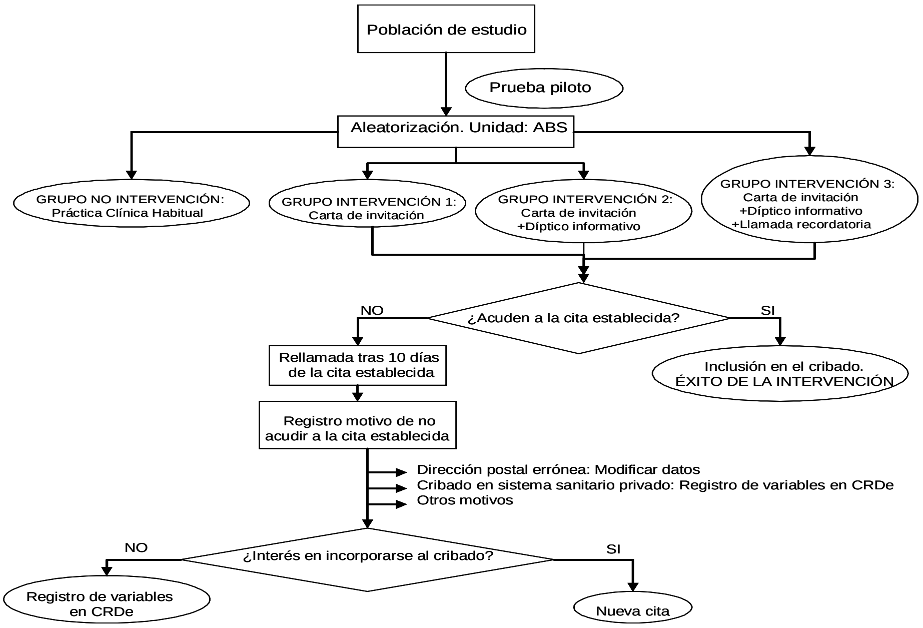

Anexo 6. CARTA DE INVITACIÓN AL CRIBADO

Apreciada Sra........

Su equipo del Centro de Atención Primaria (CAP) y de la Atención a la Salud Sexual y Reproductiva (ASSIR) , espera que tenga un año gozando de buena salud.

Es muy importante cuidar y mantener la salud, según las recomendaciones del Departamento de Salud de la Generalitat de Catalunya, especialmente en lo que hace referencia a la promoción y la prevención de las enfermedades.

El cáncer de cuello de útero es entre las mujeres, el segundo con mayor frecuencia y en nuestro país tiene una mortalidad de 3,1 casos por 100.000 mujeres y año, a pesar de ser uno de los que tiene un abordaje terapéutico y una prevención más fácil.

La realización de pruebas de cribado puede ayudar a prevenir la aparición de alteraciones o detectarlas de forma precoz, permite hacer un seguimiento más cercano y un tratamiento adecuado en las fases más iniciales de la enfermedad. Los estudios realizados demuestran que la participación de una gran parte de la población en el cribado rutinario (cada 3 años) con citología cérvico-vaginal disminuiría hasta un 80% la incidencia de este tipo de cáncer.

La citología , es una prueba, que no causa dolor ni molestia, debido a que se realiza rápidamente cogiendo una muestra a través de la vagina. Para mejorar el resultado de la prueba no hay que acudir con menstruación ni ponerse medicamentos o cremas vía vaginal los tres días previos. Esta prueba se la realizará la matrona.

Ahora nos ponemos en contacto con usted, para darle día , hora y lugar dónde le hemos reservado cita para hacerse la citología y que consta en la parte superior de la carta

Si por cualquier razón, no puede venir a la visita o ya se realiza la prueba en la sanidad privada, le agradeceríamos que contactara, lo antes posible, con la Oficina Técnica de cribaje de cáncer de cérvix, con Maribel Usart en el teléfono 93-594.21.99, para intentar encontrar la solución más adecuada.

Si tiene cualquier duda sobre la realización de la prueba o le gustaría comentar algún aspecto de esta información, se puede poner en contacto en el mismo teléfono.

Con la confianza que esta prueba le puede beneficiar, muy cordialmente.

Dr./Dra..
Dra. Amèlia Acera/ Sra. Pilar Soteras
Sra./sr..
Médico de Familia.Enfermera EAP

OFICINA TÉCNICA DE CRIBAJE DE CÀNCER DE CÉRVIX

COORDINADORAS ASSIR

Anexo 7.
PLAN DE TRABAJO PARA EL CRIBADO DE CÁNCER DE CUELLO UTERINO DE MUJERES SANAS

Objetivo: Implantar la tecnología y la periodicidad en el cribado de cáncer de cuello uterino adecuada al conocimiento científico y a la realidad de los servicios sanitarios públicos de Cataluña, de forma que se pueda conseguir una cobertura máxima sostenible de la población de riesgo.

Para llevar a cabo este objetivo, se recomienda que:

1. Mujeres de 25-65 años: toda mujer que ha iniciado las relaciones sexuales se debería incorporar al protocolo de cribado en el período de tres años desde que las inició; sería necesario que a los 25 años todas las mujeres sexualmente activas hubieran tenido la oportunidad de entrar en el protocolo de cribado de cáncer de cuello uterino.

a) Al entrar en el protocolo, se recomienda hacer dos citologías de Papanicolau con un intervalo de un año. Si las citologías son negativas, se recomienda efectuar una
cada tres años hasta los 65 años.
b) El cribado finaliza alrededor de los 65 años con una última citología a toda mujer que haya tenido un cribado adecuado y con resultados negativos.
c) Las mujeres histerectomizadas por patología benigna no deben seguir un control citológico.

2. Mujeres de 40-65 años con un cribado inadecuado: a las mujeres de 40 a 65 años que no se han hecho una citología en los cinco años previos se les ofrece un cribado con citología de Papanicolau y una prueba de determinación del VPH de alto riesgo. Si el VPH es positivo, con citología negativa, se repite la citología al cabo de 6-12 meses. Hay que repetir el VPH al cabo de un año y colposcopia si sigue positivo.

3. Mujeres de más de 65 años con un cribado inadecuado: a las mujeres de más de 65 años sin historia previa de citologías o a las cuales se hizo la última citología antes de los 60 años se les hará una citología de Papanicolau y una determinación del VPH. Si ambas pruebas son negativas, la mujer sale del protocolo.

4. Las ABS participan activamente en la identificación de mujeres mal cribadas y en la toma de muestras que hará personal formado. Este personal puede incluir llevador/as, enfermero/as o médicos/as de familia siempre que se pueda acreditar una formación adecuada en la toma de muestras. Se debe aprovechar cualquier contacto con el sistema sanitario para evaluar el estado del cribado de la mujer y actuar según las recomendaciones anteriores. Se utilizarán trípticos informativos sobre el cribado.

Las técnicas de cribado que deben considerarse son:

1. La citología cervical: se considera la prueba adecuada para la detección del cáncer de cuello uterino y sus lesiones precursoras, siempre que se sigan los controles de
calidad establecidos en todo el proceso.
2. La prueba de determinación del VHP de alto riesgo oncológico: debe cumplir unos criterios de control de calidad.

Anexo8.
HOJA INFORMATIVA DEL ESTUDIO “Evaluación Económica de tres estrategias de cribado poblacional de cáncer de cérvix en la comarca del Valles Occidental: Estudio CRICERVA”

Justificación: el cáncer cervico-uterino es el segundo más frecuente en el mundo entre las mujeres y en España tiene una mortalidad de 3,1 casos por 100.000 mujeres año, a pesar de que es una de las neoplasias de más fácil abordaje terapéutico y preventivo. Hay evidencia científica de que la participación de una gran parte de la población en el cribado rutinario disminuiría hasta un 80% la incidencia de éste tipo de cáncer.

Centros participantes: Centros de Atención Primaria y el Programa de Atención a la Salud Sexual y Reproductiva de Cerdanyola, Ripollet, Barberà y Badia del Vallès.

Desarrollo del estudio: se trata de un estudio de carácter poblacional, en el cual nos planteamos conocer la efectividad de tres tipos de intervenciones para mejorar la adherencia al cribado de cáncer de cuello uterino. Hemos incluido en el estudio a todas las mujeres de 30 a 70 años que no tengan una citología realizada en los últimos tres años. Haremos un estudio con 3 grupos intervención y un grupo control, elegidos por municipio al azar. Si usted participa en el grupo control, recibirá el seguimiento habitual que se realiza en nuestro territorio para el cribado del cáncer de cuello uterino. Si usted participa en uno de los grupos intervención, es reclutada de diferentes formas según el grupo al que haya sido asignada. A todas las participantes se les realiza una citología cervico-uterina y un cuestionario donde se recogerán datos sobre sus hábitos preventivos en relación al cáncer de cuello uterino y otros de tipo general, la mayoría de los cuales ya constan en su historia clínica. Este estudio no prevé ningún tipo de compensación económica.

Beneficios por su participación: la realización del cribado de cáncer de cuello uterino de forma correcta disminuirá el riesgo de presentar lesiones premalignas y cáncer invasivo en todas las mujeres que han respondido a la invitación al cribado. Si usted pertenece al grupo control nos ayudará a poder demostrar que nuestra hipótesis es cierta, y en caso de serlo, lo aplicaríamos también en su centro, con lo cual también se beneficiaría de las intervenciones propuestas.

Información confidencial: sus datos serán utilizados por los investigadores para su análisis. La información que se recoja sobre todos los participantes será introducida en un archivo informático, tratada confidencialmente, analizada y difundida siempre de forma agregada, de tal manera que ningún participante será identificado personalmente en la comunicación y publicación de los resultados. Toda la información personal obtenida será confidencial y no será revelada a nadie sin su permiso previo por escrito. La transmisión de datos se hará con medidas de seguridad adecuadas en cumplimiento de la “Ley Orgánica 15/1999, de 13 de diciembre de Protección de Datos de carácter personal”.

Participación voluntaria:
Su participación en este estudio es voluntaria y en cualquier momento puede retirarse del mismo. Si decide hacerlo no tendrá ninguna consecuencia en la asistencia sanitaria que usted recibirá.

Investigadores del estudio:
Si tiene alguna duda sobre el estudio o le gustaría comentar algún aspecto de esta información, por favor póngase en contacto con la Dra Amelia Acera, coordinadora del proyecto,en el teléfono …………………. o en la dirección de correo electrónica …………………

Anexo 9.
Evaluación Económica de tres estrategias de cribado poblacional de cáncer de cérvix en la comarca del Valles Occidental: Estudio CRICERVA

HOJA DE CONSENTIMIENTO INFORMADO

Yo………………………………………………………….........................(nombre y apellidos)

He leído la hoja de información que me han dado.
He podido hacer preguntas sobre el estudio.
He recibido suficiente información sobre el estudio.

He hablado con..(nombre del profesional sanitario)

Comprendo que mi participación es voluntaria.

Comprendo que puedo retirarme del estudio:
Cuando quiera.
Sin tener que dar explicaciones.
Sin que esto repercuta en mi atención médica.

Doy libremente mi conformidad para participar en el estudio en el día de hoy,

En..el..................de.......................... del 20......

....	....	....
Nombre y apellidos del participante	Firma	Fecha
....	....	....
Nombre y apellidos del profesional sanitario	Firma	Fecha

Eliminado: – – – Salto de página– – –

Con formato: Numeración y viñetas
