# Supplementary material for: Increasing Cervical Cancer Screening Coverage: A Randomised, Community-Based Clinical Trial
Source: PLoS One. 2017 Jan 24;12(1):e0170371. doi: 10.1371/journal.pone.0170371 (PMC5261609; doi:10.1371/journal.pone.0170371)
Supplement: S5 File — (PDF) [file pone.0170371.s005.pdf]

BMC Public Health

Cost-effectiveness of strategies to increase screening coverage for cervical cancer in Spain: the CRIVERVA study

--Manuscript Draft--

**Manuscript Number:**	PUBH-D-16-01311R1
**Full Title:**	Cost-effectiveness of strategies to increase screening coverage for cervical cancer in Spain: the CRIVERVA study
**Article Type:**	Research article
**Section/Category:**	Health policies, systems and management in high-income countries
**Funding Information:**	Ministerio de Sanidad, Servicios Sociales e Igualdad (PI10/01275) — Ms Amelia Acera Pérez<br>Instituto de Salud Carlos III (RTIC RD06/0020/0095; RD12/0036/0056) — Ms Amelia Acera Pérez<br>Agència de Gestió d’Ajuts Universitaris i de Recerca (2014SGR1077; 2014SGR2016) — Ms Amelia Acera Pérez
**Abstract:**	Background<br>The aim of this study it to carry out a cost-effectiveness analysis of three different interventions to promote the uptake of screening for cervical cancer in general practice in the county of Valles Occidental, Barcelona, Spain.<br>Methods<br>Women aged from 30 to 70 years (n=15,965) were attracted to attend a general practice to be screened. They were randomly allocated to one of four groups: no intervention group (NIG); one receiving an invitation letter to participate in the screening (IG1); one receiving an invitation letter and informative leaflet (IG2); and one receiving an invitation letter, an informative leaflet and a phone call reminder (IG3). Clinical effectiveness was measured as the percentage increase in screening coverage. A cost-effectiveness analysis was performed from the perspective of the public health system with a time horizon of 3,5 years, the duration of the randomized controlled clinical trial. In addition, a deterministic sensitivity analysis was performed. Results are presented according to different age groups.<br>Results<br>The Incremental Cost-Effectiveness Ratio (ICER) for the most cost-effective intervention, IG1, compared with opportunistic screening was 2.78€ per one percent increase in the screening coverage. The age interval getting worst results in terms of efficiency was for women aged <40 years.<br>Conclusions<br>In a population like Catalonia with around 2 million women aged 30 70 years and assuming that 40% of these women were not attending general practice to be screened for cervical cancer, the implementation of a intervention to increase the screening coverage that consists in sending a letter would cost on average less than 490€ for every 1,000 women.
**Corresponding Author:**	Marta Trapero-Bertran, PhD<br>Universitat Internacional de Catalunya<br>Barcelona, Barcelona SPAIN
**Corresponding Author Secondary Information:**	
**Corresponding Author's Institution:**	Universitat Internacional de Catalunya
**Corresponding Author's Secondary Institution:**	
**First Author:**	Marta Trapero-Bertran, PhD
**First Author Secondary Information:**	
**Order of Authors:**	Marta Trapero-Bertran, PhD

Powered by Editorial Manager® and ProduXion Manager® from Aries Systems Corporation

	Amelia Acera Pérez
	Silvia de Sanjosé
	Josep Maria Manresa Domínguez
	Diego Rodríguez Capriles
	Ana Rodríguez Martínez
	Josep Maria Bonet Simó
	Norman Sanchez Sanchez
	Pablo Hidalgo Valls
	Mireia Díaz Sanchis
**Order of Authors Secondary Information:**	
**Response to Reviewers:**	Barcelona, 5th August 2016<br><br>Dear Editors,<br>We are pleased to submit a revision of the article entitled " Cost-effectiveness of strategies to increase screening coverage for cervical cancer in Spain: the CRIVERVA study". Thank you very much for giving us the opportunity to address all the reviewers' comments. We are very grateful to the two reviewers for their helpful comments that surely have helped to improve this manuscript.<br>In response to comments from Reviewer 1:<br>1.Authors need to make substantial language editing and corrections of the entire manuscript including correction of many typographical errors (lines 59,81,122,127,131,136,176,179,221,224,230,251).<br>Thank you so much to reviewer 1 for this comment. We have reviewed the whole manuscript again and we have contracted some professional editing services (Elsevier editing services, please find enclosed the certificate of it) that have gone through the entire manuscript.<br>2.In addition, the authors should maintain consistency in currency for ease of understanding e.g. lines42 and 43 of the manuscript where 'A$' was interchangeably used with '€'.<br>Thank you very much for this comment. This has been amended (see line 42).<br><br>In response to comments from Reviewer 2:<br>1.The first comment is to indicate that the manuscript requires extensive copy-editing. The grammatical and spelling errors are too numerous to itemize.<br><br>Thank you so much to reviewer 2 for this comment. We have reviewed the whole manuscript again and we have contracted some professional editing services (Elsevier editing services, please find enclosed the certificate of it) that have gone through the entire manuscript.<br><br>2.In the Methods section, Authors have obviously not clearly described the current study sufficiently to distinguish it from the CRICERVA Project. The study is apparently nested within this larger study. The full details of the study need to be explained. For example, one only got to know that they conducted an interview with the aid of a questionnaire only in Table 3.<br><br>Thank you so much for this comment. Full details of the trial have been incorporated in the main text (see Methods section – CRICERVA study, please, line 81).<br><br>3.Page 7. Last sentence: Authors to review ....'to undergo' what?<br>Thank you so much for this comment. Full details of the trial have been incorporated in the main text (see<br>Thank you so much for this comment. This sentence has been rephrased (please, check from line 124 onwards).<br>4.What informed the different sample size for the intervention and control arms?<br>Thank you so much for this comment. Full details of how the sample was calculated has been added within the main text (please, check from line 96 onwards).

	5.Table 1: Answer to the intervention'. Do Authors intend to report the response to the invitation or something similar?<br>Thank you so much for this comment. A note to Table 1 has been added for clarification.<br>6.In the Discussion section, Authors have employed two different citation styles.<br>Page 12. The last sentence: '..........would cost 0.45E for woman (392,000E)'is unclear.<br>Thank you so much for this comment. References have been cited numerical all across the discussion section.<br>7.When was Reference 1 accessed?<br>Thank you so much for this comment. Reference 1 has been completed as required.<br>8.In Reference 10, which BMC journal is being referred to?<br>Thank you so much for this comment. Reference 10 has been completed as required.<br><br><br>With thanks in advance for your help and looking forward to hearing from you.<br><br>Yours sincerely,<br>Marta Trapero-Bertran MSc PhD (on behalf of all authors)

Marta Trapero-Bertran
Universittat Internacional de Catalunya (UIC)
Faculty of Economic and Social Sciences
Immaculada 22
08017 Barcelona
Spain

Barcelona, 5th August 2016

Dear Editors,

We are pleased to submit a revision of the article entitled " Cost-effectiveness of strategies to increase screening coverage for cervical cancer in Spain: the CRIVERVA study". Thank you very much for giving us the opportunity to address all the reviewers' comments. We are very grateful to the two reviewers for their helpful comments that surely have helped to improve this manuscript.

In response to comments from Reviewer 1:

1. *Authors need to make substantial language editing and corrections of the entire manuscript including correction of many typographical errors (lines 59,81,122,127,131,136,176,179,221,224,230,251).*

Thank you so much to reviewer 1 for this comment. We have reviewed the whole manuscript again and we have contracted some professional editing services (Elsevier editing services, please find enclosed the certificate of it) that have gone through the entire manuscript.

2. *In addition, the authors should maintain consistency in currency for ease of understanding e.g. lines42 and 43 of the manuscript where 'A$' was interchangeably used with '€'.*

Thank you very much for this comment. This has been amended (see line 42).

In response to comments from Reviewer 2:

1. *The first comment is to indicate that the manuscript requires extensive copy-editing. The grammatical and spelling errors are too numerous to itemize.*

   Thank you so much to reviewer 2 for this comment. We have reviewed the whole manuscript again and we have contracted some professional editing services (Elsevier editing services, please find enclosed the certificate of it) that have gone through the entire manuscript.

2. *In the Methods section, Authors have obviously not clearly described the current study sufficiently to distinguish it from the CRICERVA Project. The study is apparently nested within this larger study.*

The full details of the study need to be explained. For example, one only got to know that they conducted an interview with the aid of a questionnaire only in Table 3.

Thank you so much for this comment. Full details of the trial have been incorporated in the main text (see Methods section – CRICERVA study, please, line 81).

3. *Page 7. Last sentence: Authors to review ....'to undergo' what?*

Thank you so much for this comment. Full details of the trial have been incorporated in the main text (see

Thank you so much for this comment. This sentence has been rephrased (please, check from line 124 onwards).

4. *What informed the different sample size for the intervention and control arms?*

Thank you so much for this comment. Full details of how the sample was calculated has been added within the main text (please, check from line 96 onwards).

5. *Table 1: Answer to the intervention'. Do Authors intend to report the response to the invitation or something similar?*

Thank you so much for this comment. A note to Table 1 has been added for clarification.

6. *In the Discussion section, Authors have employed two different citation styles. Page 12. The last sentence: '..........would cost 0.45E for woman (392,000E)'is unclear.*

Thank you so much for this comment. References have been cited numerical all across the discussion section.

7. *When was Reference 1 accessed?*

Thank you so much for this comment. Reference 1 has been completed as required.

8. *In Reference 10, which* BMC *journal is being referred to?*

Thank you so much for this comment. Reference 10 has been completed as required.

With thanks in advance for your help and looking forward to hearing from you.

Yours sincerely,

Marta Trapero-Bertran MSc PhD (on behalf of all authors)

Manuscript
Click here to download Manuscript BMC_CRICERVA CEA_FIN_MTB_050816_elsevier edited.doc
Click here to view linked References

Cost-Effectiveness of Strategies to Increase Screening Coverage for Cervical Cancer in Spain: The CRIVERVA Study

Marta Trapero-Bertran,[1,2] mtrapero@uic.es, Amelia Acera Pérez,[3,4,5,6] amelia.acera@gmail.com, aacera.mn.ics@gencat.cat, de Sanjosé S,[7] desanjose.silvia@gmail.com, Josep Maria Manresa Domínguez,[4,8] jmanresa@idiapjgol.info, Diego Rodríguez Capriles[3] rodriguez.capriles@gmail.com, Ana Rodriguez Martinez,[3] arodriguezma.mn.ics@gencat.cat, Josep Maria Bonet Simó,[9] jmbonets@gencat.cat, Norman Sanchez Sanchez,[10] normansanchez@gencat.cat, Pablo Hidalgo Valls,[9] cbarbera.mn.ics@gencat.cat, and Mireia Díaz Sanchis,[7] mireia@iconcologia.net

Authors Information

[1] Economy and Business Organization Department, Faculty of Economics and Social Sciences. Universitat Internacional de Catalunya (UIC), Barcelona, Spain.

[2] Center for Research in Economics and Health (CRES), University Pompeu Fabra, Barcelona, Spain.

[3] Atenció a la Salut Sexual i Reproductiva (ASSIR) SAP Cerdanyola—Ripollet, Institut Catala de la Salut, Ripollet, Barcelona, Spain

[4] Unitat de Suport a la Recerca Metropolitana Nord, IDIAP Jordi Gol. Sabadell, Barcelona, Spain

[5] Grup de Recerca GRASSIR reconegut per la Generalitat de Catalunya SGR 2014-2016

[6] Universitat de Barcelona, Spain

[7] Unit of Infections and Cancer, Institut Català d'Oncologia, L'Hospitalet de Llobregat, Barcelona, Spain

[8] Departament de Infermeria, Universitat Autonoma de Barcelona, Bellaterra, Barcelona, Spain

[9] SAP Vallés Occidental, Institut Català de la Salut, Sabadell, Barcelona, Spain

[10] Sistemes d'Informació Sanitària, SAP Vallés Occidental, Institut Catala de la Salut, Sabadell, Barcelona, Spain

Corresponding Aauthor

Marta Trapero-Bertran, (mtrapero@uic.es)

c/Immaculada 22

08017 Barcelona,, Spain.

Tel.f: +34 932 541 800

mtrapero@uic.es

Abstract

Background

The aim of this study is~~t~~ to carry out a cost-effective~~ness~~ analysis of three different interventions to promote the uptake of screening for cervical cancer in general practice in the county of Valles Occidental, Barcelona, Spain.

Methods

Women aged from 30 to 70 years (n=15,965) were ~~attracted~~ asked to attend a general practice to be screened. They were randomly allocated to one of four groups: no intervention group (NIG); one receiving an invitation letter to participate in the screening (IG1); one receiving an invitation letter and informative leaflet (IG2); and one receiving an invitation letter, an informative leaflet and a phone call reminder (IG3). Clinical effectiveness was measured as the percentage increase in screening coverage. A cost-effectiveness analysis was performed from the perspective of the public health system with a time horizon of 3 to ~~,~~5 years, the duration of the ~~randomized~~ randomised controlled clinical trial. In addition, a deterministic sensitivity analysis was performed. Results are presented according to different age groups.

Results

The incremental cost-effectiveness ratio (ICER) for the most cost-effective intervention, IG1, compared with opportunistic screening was 2.78€ per ~~one percent~~1% increase in the screening coverage. The age interval getting the worst results in terms of efficiency was ~~for~~ women aged < 40 years.

Conclusions

In a population like Catalonia, with around 2 million women aged 30 to 70 years and assuming that 40% of these women were not attending general practice to be screened for cervical cancer, the implementation of an intervention to increase the screening coverage that consists ~~in~~ of sending a letter would cost on average less than 490€ for every 1,000 women.

Trial registration: ClinicalTrials.gov Identifier: NCT01373723

Formatted: English (United Kingdom)

Key-words:

cCost-effectiveness, population screening, cervical cancer, and, increase coverage.

Formatted: Space After: 0 pt

Background

In Spain, both cervical cancer incidence and survival have remained stable in the p~~l~~ast few years (de Sanjosé and Garcia 2006 and Allemani 2014). The global estimate of the age-adjusted incidence rate of invasive cervical cancer was 7.8 per 100,000 woman-years in 2012,[1] ~~[1]~~ which means that Spain is in the low-mid range of the European countries (3.6-28.6 per 100.000 woman-years). In the Autonomous Region of Catalonia, the truncated incidence rate is 16.1 per 100,000 woman-years for those aged from 35 to 64 years, being the risk of developing a cervical cancer one per each 106 women who have lived until~~l~~ 75 years old, ~~[2]~~.[2] The 5-year net survival in Spain was 65.2 for women diagnosed during 2005-2009 and comparable or even higher than most developed countries. Despite these relatively positive data, cervical cancer is still a public health concern because is largely preventable and also ~~for~~ due to the high cost of screening and treatment of cervical lesions.

Cancer cost the EU €126 billion in 2009, with health care accounting for €51·0 billion (40%).[3] ~~[3].~~ In Australia, with lower cervical cancer incidence and higher survival than Spain, the total cost of the screening programme was estimated to be 130.4M € (2015) and the treatment cost accounted for approximately one-~~-~~third of the total (109,8M € [~~(~~2015]~~)~~).[4] ~~[4].~~

In Spain, cytological screening for cervical cancer is largely opportunistic with some variations in the protocol according to the region ~~[5]~~.[5] Eighty percent of the cases of cervical cancer in Catalonia have not undergone previous cytology during the 10 years prior to diagnosis ~~[6]~~.[6] In Catalonia, the protocol, which was revised and modified by the Oncology Director Plan and the Catalan Institute of Oncology in 2006, ~~did~~ incorporated the establishment of triennial periodicity of cytologies in women from 25 to 65 years of age~~;~~ and~~,~~ ~~the incorporation~~ of the HPV test in women from 40 to 65 years of age with no prior cytology within the previous 5 years or with a cytology carried out for longer than 5 years, abnormal cytology (no specified atypical squamous lesions), and women with post-~~conization~~ conisation control of intraepithelial lesions. An increase in screening coverage through interventions promoting the uptake of screening should be a priority objective for health care authorities if cervical

Formatted: English (United Kingdom), Superscript, Kern at 11 pt

Formatted: English (United Kingdom)

Formatted: English (United Kingdom), Superscript, Kern at 11 pt

Formatted: English (United Kingdom)

Formatted: English (United Kingdom), Superscript, Kern at 11 pt

Formatted: English (United Kingdom)

Formatted: English (United Kingdom)

Formatted: English (United Kingdom), Highlight

Formatted: English (United Kingdom)

Formatted: English (United Kingdom), Superscript, Kern at 11 pt

Formatted: English (United Kingdom)

Formatted: English (United Kingdom)

cancer cases are to be reduced and women who do not periodically have~~d~~ a cytology are to be identified.

According to a systematic review of the Cochrane collaboration[7] ~~[7]~~ evaluating interventions to stimulate the participation of women in the screening of this disease, invitations and educational interventions seem to be the most effective ways to increase the participation in screening programmes. In addition, there is sufficient evidence of increasing coverage when using ~~individualized~~ individualised information directed to the target population, especially with systems for call-recall (that is~~i.e.,~~ ~~sms~~SMS, email, phone calls) ~~[8-9]~~.[8-9] Forbes et al. encourages providing trials to further support strategies to increase coverage ~~[7]~~.[7] This would facilitate earlier action in detecting pre-malignant lesions, helping to reduce the incidence of invasive cancer and their costs. Therefore, there is a need for evaluating strategies to increase the screening population coverage for the efficiency ~~point of view~~. This will allow decision makers to better inform decisions on which preventive programmes to conduct in Spain. The CRICERVA study[10]~~]~~ is a cluster clinical trial ~~which~~ that assigned ~~one~~ 1 of ~~three~~ 3 interventions to the target population~~,~~ registered in the Cerdanyola SAP area in Barcelona. A total of 32,858 women residing in the study area and~~,~~ aged 30 to 70 years and with no record of cervical cytology during the past 3.5 years were selected. The study included 4 arms: 3 interventions (a ~~personalized~~ personalised invitation letter, an informative leaflet added, and a ~~personalized~~ personalised phone call added) and a control group (based on spontaneous demand).

The aim of this study is to perform a cost-effectiveness anal~~y~~isis~~,~~ alongside the CRICERVA clinical trial,[10]~~],~~ of ~~three~~ 3 different active interventions to promote the uptake of screening for cervical cancer in general practice. An orientative protocol of this economic evaluation was first published in 2011.[11]~~].~~

Formatted: English (United Kingdom)

Formatted: English (United Kingdom)

Formatted: Superscript, Kern at 11 pt

Formatted: English (United Kingdom), Superscript, Kern at 11 pt

Formatted: English (United Kingdom)

Formatted: English (United Kingdom)

Formatted: English (United Kingdom)

Formatted: English (United Kingdom)

Formatted: English (United Kingdom), Superscript, Kern at 11 pt

Formatted: English (United Kingdom)

Formatted: English (United Kingdom), Superscript, Kern at 11 pt

Formatted: English (United Kingdom)

Methods

CRICERVA Pproject

The CRICERVA Study was a cCommunity-based cluster clinical trial, with 4 arms assigned by groups and performed in a predefined geographical area, from the Primary Health Care Service (SAP) Cerdanyola, in the metropolitan belt of Barcelona, Spain, and it was subdivided into 5 study areas, 4 of which were included in this study. SAP Cerdanyola covered a population of 120,293 individuals over the age of 14 years. The fFemale population aged between 30-70 ascribed to the study areas were: study area-1: N=8,968; study area-2: N=8,169; study area-3: N=11,027; and, study area-4: N=4,694. For the study purposes, eligibility included women aged between 30 to 70 years of age, and whose general practitioner was ascribed to the SAP Cerdanyola area, were residents in of the area for more than 6 months, and with had no record in the medical registry of screening of cervical cancer in the prior 3.5 years. This resulted in the identification of 15,965 out of 32,858 (48.58%) women. Selected women were clustered randomlyized and contacted according to the allocated arm. When the personal contact was established, they were asked for the acceptance to answer the interview. The interview allowed us to identify those women susceptible to be screened and invite them for screening.

The sample size was calculated based on the detection of a difference in effectiveness compared with the non intervention group (NIG). It has been calculated by multiplying the size of a simple randomised design by the design effect or factor of inflation. For the simple randomised design, on accepting an alpha risk of 0.05 and a beta risk of 0.20 in a bilateral contrast, 59 subjects were required in the first group and 59 in the second group to detect a difference greater than or equal to 28.4% in the screening coverage of the 41.6% in the NIG. The lost to follow--up rate was estimated at 20%. The calculation of the sample was

Formatted: English (United Kingdom), Highlight

performed with the Granmo 5.2 computer programme for Windows. Considering an intraclass correlation coefficient of 0.05 and a mean number of 3,500 women from 30 to 70 years of age with incorrect screening by Basic Health Care Area, the design effect was 176 and thus, 20,768 women with incorrect screening ~~was~~ were required. Women eligible for screening were verbally informed about the screening procedures and the significance of the results. Women were excluded if they had ~~and~~ a hysterectomy, ~~those with~~ a current history of cervical intraepithelial lesions, carcinoma in situ and cervical uterine cancer, ~~had~~ a diagnosis of HIV or ~~of~~ immunosuppression. All members of the targeted population ~~was~~ were invited to participate. ~~S~~AP Cerdanyola was divided into 5 Basic Health Care Centers (BHCC), 4 of which were included in the study.

Briefly, the cluster unit was each of 4 BHCC. Each of the 4 participating BHCC were randomly assigned to one study arm. The follow-~~-~~up period of this trial finished when the diagnosis of each screening visit was completed. After ~~having~~ completing~~ed~~ the recruitment of the intervention groups, we ~~proceed to characterized~~ characterised the women in the NIG in terms of screening practices and, if adequate, invited~~ing~~ them to be screened.

Interventions evaluated were 1) a ~~personalized~~ personalised invitation letter to participate in the screening signed by the patient's primary care physician and professionals of the corresponding Public Health Center (IG1); 2) the same letter of invitation sent in the IG1 as well as an informative leaflet on the prevailing screening of cervical cancer (IG2); and~~,~~ 3) the same intervention as the one performed in IG2, complemented by a phone call 3 days prior to the appointment indicated in the letter of invitation as a reminder of the visit (IG3). These three interventions were compared to the ~~no intervention group~~NIG with current opportunistic screening ~~(NIG)~~. There was one common action in the three different interventions, which was scientifically validated as effective, consisting ~~in~~ of a ~~personalized~~ personalised invitation letter sent by the primary health care professionals including a fixed appointment with the GP to get a cytology test, and ~~other~~ two other different interventions

Formatted: Highlight
Formatted: English (United Kingdom), Highlight
Formatted: Highlight
Formatted: English (United Kingdom), Highlight
Formatted: Highlight
Formatted: English (United Kingdom), Highlight
Formatted: Highlight
Formatted: English (United Kingdom), Highlight

(informative leaflet and reminder call) to evaluate approaches for which there are few studies assessing the effectiveness of attendance to screening programmes. Women were distributed into 4,197 patients to IG1, 3,601 to IG2, 6,088 to IG3, and 2,079 to the NIG. Sociodemographic characteristics of the population ~~could be consulted on~~are seen in Table 3. From ~~all~~ these, 1,~~.~~377 (47%) women in IG1, 1,~~.~~258 (48 %) in IG2, and 1,628 (40%) ~~women~~ in IG3 did not meet the appointments. In addition, 1,~~.~~248 women in IG1, 976 in IG2, and 2,064 in IG3 were excluded because of an adequate screening private system, a~~n~~ hysterectomy, a cervical disease, a change of address, or death. These numbers ~~end~~ add up to 1,578 screening visits in IG1, 1,367 visits in IG2, and 2,396 visits in IG3. Hence, the average total number of patients ~~that~~ who responded to all the interventions was approximately 56%. The highest ~~respond~~ response rate was observed in the IG2 group (58.3%), followed by IG1 (55.9%), and the IG3 (53.7%). The youngest (younger than 40 years) and the elderly (~~equal or older than~~ 70 years or older) were the groups ~~less~~ least ~~responding~~ responsive to any intervention. Table 1 shows the target population; women invited to participate in this study because ~~of~~ the last screening happened before ~~of~~ three and a half years ~~ago~~prior; women who were contacted and were willing to attend the GP visit; and the~~,~~ number of women who finally attended the GP visit.

. The Ethical Committee of the Institute of Research in Primary Care (IDIAP Jordi Gol) from Catalonia, Spain, approved this study, ~~same~~ as well as the CRICERVA study.[10]~~.~~

Health Outcome and Cost~~ing~~ Data

Effectiveness data ~~was~~ were provided from the CRIVERVA project.[10]~~.~~ The outcome measure was the increase in the screening coverage over 42 months. The acceptance rate was highest among the ~~group~~ IG3 group (23%), followed by IG1 (18.6%), while ~~the intervention~~ IG2 ~~was the one with~~had the lowest average success rate (17.4%).

Formatted: English (United Kingdom), Check spelling and grammar

Formatted: English (United Kingdom), Superscript, Kern at 11 pt

Formatted: English (United Kingdom)

Formatted: English (United Kingdom)

Formatted: English (United Kingdom), Superscript, Kern at 11 pt

Formatted: English (United Kingdom)

The analysis was performed from the Public Health System perspective and therefore only direct health care costs were included. All available management costs per unit were adapted from Diaz et al.[12] whereas strict costs from interventions were calculated from the Reproductive and Sexual Health Primary Care Unit (ASSIR)[13] (~~see~~ Table 2). Management costs included ~~fifteen~~ 15 minutes of a nurse or midwife visit, a citology for taking the smear, and an ~~hpv~~ HPV test. Inflation rates had been applied to get management costs in 2014.[14] These three costs were considered in the three interventions and also for the NIG ~~no intervention group~~ because all women coming or not opportunistically to the Basic Health Care Area (BHCA) were ~~incoming in~~incurring these costs. However, the costs for each of the interventions were different. The IG1 included the costs of a letter, its posting, and ~~two~~ 2 minutes of an officer's time to prepare the posting. The IG2 considered also the costs of IG1 plus a leaflet and just some seconds more of the officer's time to prepare this posting. Finally, the IG3~~,~~ considered not only the costs of IG2 but the cost of a reminding call, lasting ~~between one~~ 1 ~~and~~ to ~~five~~ 5 minutes, and the extra officer time spent on it. Costs were expressed in € 2014.

Anal~~i~~ysis

The time horizon of the analysis was 3.5 years, the duration of the ~~randomized~~ randomised controlled clinical trial. Costs and effects were not discounted because the results are reported over the trial period. A cost-effectiveness analysis of the different interventions was performed using incremental cost-effectiveness ratios (ICERs).[15]

~~The~~ ICERs were calculated as the additional benefit to be gained in € per effectiveness unit (1% coverage) from an alternative compared to another.

$$\frac{\text{Difference in Costs Between two Interventions}}{\text{Difference in the \% of Screening Coverage Between two Interventions}}$$

Formatted: English (United Kingdom), Superscript, Kern at 11 pt
Formatted: English (United Kingdom)
Formatted: English (United Kingdom), Superscript, Kern at 11 pt
Formatted: English (United Kingdom)
Formatted: English (United Kingdom), Superscript, Kern at 11 pt
Formatted: English (United Kingdom)
Formatted: English (United Kingdom)
Formatted: English (United Kingdom), Superscript, Kern at 11 pt
Formatted: English (United Kingdom)
Formatted: English (United Kingdom), Check spelling and grammar
Formatted: English (United Kingdom)

All results were presented according to different age groups (< 40; 40-49; 50-59; ≥60). In order to measure the uncertainty of results, a deterministic univariate sensitivity analysis was performed to examine the effect of the uncertainty o~~i~~n the effectiveness parameter.

Results

Table 3 describes the sociodemographic and behavioural characteristics of women interviewed in each intervention group. Table 4 presents the cost-effectiveness analysis of the interventions groups. The ICERs~~,~~ competing choices approach~~,~~ shows that including women of all ages, IG2 is strongly dominated because is more expensive and less effective than IG1. IG1 costs €2.78~~€~~ per 1% increase in coverage compared to an opportunistic screening and IG3 costs €13.73~~€~~ per 1% increase in coverage more than an opportunistic screening, ~~being the~~making IG1 more cost-effective. In the comparisons with the next best alternative, IG3 costs €60.73~~€~~ per 1% increase in coverage more than IG1. Therefore, for women of all age~~'~~s, ~~women~~ IG1 is the most cost-effective alternative. Results differ in scale across age groups but not conceptually, and IG2 is always strongly dominated by IG1 (see Table 5). ICERs for IG1, compared to the opportunistic screening or the next best alternative, are lower than €4~~€~~ per 1% increase in coverage for all age ~~-~~groups. The IG2 costs €103.85~~€~~ per 1% increase in coverage more than IG1 for women ≥60 years;~~,~~ for the rest of the age groups, IG2 compared to IG1 is either a dominated or a more expensive alternative.~~.~~ The age group obtaining worst results in terms of efficiency was women aged <40 years, although ICERs are still quite economically sensible (€3.55~~€~~ per 1% increase in coverage for IG1 and €177.86~~€~~ per 1% increase in coverage for IG3). Therefore, consistently, ~~the intervention of~~ sending a letter seems to be the most cost-effective intervention~~s~~ for all ages of women~~ ages~~.

Sensitivity Analysis

When the increase of coverage is reduced by 50%, results remain the same in terms of efficiency ranking, with the option of sending a letter (IG1) being the most cost-effective intervention compared with doing nothing. Even if the final coverage ~~would have~~ was ~~been~~ decreased 75% of the results experienced in the CRICERVA study, cost-effectiveness results would have remained, showing the robustness of this analysis and the low values obtained for ~~the~~ ICERs of each intervention compared with doing nothing.

Discussion

This economic evaluation evaluated whether the increase i~~o~~n participation rates of screening for cervical cancer compensates for the costs incurred from different interventions accrued. Observing our results, if a universal strategy is applied for all age-groups, the preventive intervention of sending a letter for an appointment is the most efficient with a cost around €3~~€~~ per 1% coverage, followed by sending a letter with a leaflet and a remain~~d~~ing call with a cost €61~~€~~ per 1% coverage. The intervention of sending a letter with a leaflet (IG2) is more expensive and less effective than only sending a letter (IG1). Results by age are consistent;~~,~~ the intervention of sending a letter costs less than €4~~€~~ per 1% coverage and sending a letter with a leaflet and a remain~~d~~ing call costs between €2~~€~~ and €178~~€~~ depending on the age; the older the women, the more cost-effective ~~is~~ this intervention.

Some authors already studied the cost-effectiveness of interventions to promote cervical cancer.[16]~~].~~ Although not all the interventions were the same as the ones analysed in this paper, the letter was common to all of them and the comparator was the opportunistic screening. In that paper, the most cost-effective intervention was to remind a doctor to offer a smear during a consultation;~~,~~ however, they were operating in a relatively disadvantaged area and populations are not comparable. However, ——— some authors reinforce the results we obtained in this study.[17]~~].~~ They stated that telephone contact with women who have abstained from cervical cancer screening for long time increases participation and leads to a significant increase in detection of atypical smears. Other authors also support~~s~~ the idea that contacting women through a mail reminder was as effective as, and less expensive than, a telephone call.[18]~~].~~ In our case, there was no intervention involving an email, but IG3 involved a telephone call and was the least cost-effective intervention compared to opportunistic screening.

According to some authors, there are large variations in cervical cancer screening policies, coverage, and quality of screening across Europe.[19]~~].~~ As others assessed ~~assessed~~, the recommendations of the Council of the European Union (EU) on ~~organized~~ organised population-based screening for cervical cancer ~~are~~ have not yet been fulfilled.[20]~~].~~ The European cervical cancer screening guidelines were

Formatted: English (United Kingdom), Highlight

Formatted: English (United Kingdom), Superscript, Kern at 11 pt, Highlight

Formatted: English (United Kingdom), Highlight

Formatted: English (United Kingdom), Superscript, Kern at 11 pt, Highlight

Formatted: English (United Kingdom), Highlight

Formatted: English (United Kingdom), Superscript, Kern at 11 pt, Highlight

Formatted: English (United Kingdom), Highlight

Formatted: English (United Kingdom), Superscript, Kern at 11 pt, Highlight

Formatted: English (United Kingdom), Highlight

Formatted: Highlight

Formatted: English (United Kingdom), Highlight

Formatted: English (United Kingdom), Superscript, Kern at 11 pt, Highlight

Formatted: English (United Kingdom), Highlight

prepared for all European countries (not only for EU members), but many of them failed in implementation.[21] Spain has opportunistic screening implemented by regions and the age range is established between 30 and 65 years.[19] Decisions on the target age group and frequency of screening are usually made at the national level;~~,~~ however, continued unavailability of population~~-~~based, systematically ~~organized~~ organised screening programmes to women who may benefit from screening remains ~~to be~~ the major obstacle in control of cervical cancer in Europe. Some authors claimed that evaluation of screening activity on cervical cancer using cohort studies designs among screening populations~~,~~ are proceeding in some countries, but results were not available yet.[22] Others recently stated that a shift from ~~the~~ opportunistic to ~~organized~~ organised screening is imperative to ~~optimize~~ optimise cost and impact of screening, but no~~t~~ evidence on cost-effectiveness has been published on this type of ~~studies~~ study.[23] This paper tries to cover these gaps by providing information on efficiency of different interventions to start building a national ~~organized~~ organised screening programme. However, the available evidence supports the hypothesis that while ~~organized~~ organised population screening programmes are successful in increasing overall participation rates, they may not per se substantially reduce social inequalities.[24]

With regard to the factors influencing participation in screening, some authors have suggested the following: the absence of population~~al~~ programmes; low ~~sensitization~~ sensitisation with respect to preventive attitudes in cohorts of elderly women; and~~,~~ health care overload in primary care centres [8,25].

This economic evaluation just covers the diagnosis on the illness pathway; however, this will influence the cost-effectiveness of the whole cervical cancer pathway. Therefore, there is a need to build a model for the natural history of cervical cancer for Spain, such the one built for Germany,[26] and study the cost-effectiveness ~~for~~ of the whole pathway accounting for ~~organized~~ organised cervical cancer screening programmes.

In a population like Catalunya with around 2 million women aged 30-70 years and assuming that 40% (800,~~.~~000) of these women have not been screened for the last 3 years, the implementation of a

Formatted: English (United Kingdom), Superscript, Kern at 11 pt, Highlight
Formatted: English (United Kingdom), Highlight
Formatted: English (United Kingdom), Superscript, Kern at 11 pt, Highlight
Formatted: English (United Kingdom), Highlight
Formatted: Highlight
Formatted: English (United Kingdom), Highlight
Formatted: Highlight
Formatted: English (United Kingdom), Highlight
Formatted: English (United Kingdom), Superscript, Kern at 11 pt, Highlight
Formatted: English (United Kingdom), Highlight
Formatted: Highlight
Formatted: English (United Kingdom), Highlight
Formatted: Highlight
Formatted: English (United Kingdom), Highlight
Formatted: English (United Kingdom), Superscript, Kern at 11 pt, Highlight
Formatted: English (United Kingdom), Highlight
Formatted: Highlight
Formatted: English (United Kingdom), Highlight
Formatted: Highlight
Formatted: English (United Kingdom), Highlight
Formatted: Highlight
Formatted: English (United Kingdom), Highlight
Formatted: Highlight
Formatted: English (United Kingdom), Highlight
Formatted: English (United Kingdom), Superscript, Kern at 11 pt, Highlight
Formatted: English (United Kingdom), Highlight
Formatted: English (United Kingdom)
Formatted: English (United Kingdom)
Formatted: English (United Kingdom), Superscript, Kern at 11 pt
Formatted: English (United Kingdom)
Formatted: English (United Kingdom), Superscript, Kern at 11 pt
Formatted: English (United Kingdom)
Formatted: English (United Kingdom)
Formatted: English (United Kingdom)

intervention to increase the screening coverage in 1% ~~-~~will imply that the government should be willing to pay €2.78~~€~~ (for a 1% of increase in the coverage).~~.~~ In this study, the NIG (n=428 women spontaneous demand) cost ~~to~~ the Catalonian g~~G~~overnment €36,~~.~~864~~€~~; the women attending the screening because of the letter (IG1, n=1,578) cost €136,~~.~~683~~€~~ (which increased the coverage of the screened population ~~in a~~by 17.6%); the women attending the screening because of the letter and the leaflet (IG2, n=1367) cost €120,~~.~~255~~€~~ (which increased the coverage of the screened population by ~~in~~ only 16.7%); and~~,~~ the women attending the screening because of the letter, leaflet, and phone call (IG3, n=2,396) cost €213,~~.~~484~~€~~ (which increased the coverage of the screened population ~~in a~~by 21.7%). However, if all women would have been contacted using the IG1, the most cost-effective strategy, the screening of the 5,~~.~~669 women would have cost €491,049~~€~~, therefore, the Catalonian government would have saved €16,237~~€~~. Obviously, the higher the number of women screened, the higher would be the saving. Thus, to ~~attend~~ test5,~~.~~669 women in total ~~it~~ costs €507,~~.~~286~~€~~.

Conclusion

The ICER for the most cost-effective intervention, IG1, compared with opportunistic screening was €2.78~~€~~ per ~~one percent~~1% increase in the screening coverage, being for IG2 and IG3 the elderly group (≥60) the one that gets more efficiency across interventions. Sending a letter would cost on average around €490~~€~~ for every 1,000 women and sending a letter with a leaflet. The age interval getting worst results in terms of efficiency was for women aged <40 years. This analysis encourages including this intervention in the national policy on screening to prevent cervical cancer, so this would complement the opportunistic system; meanwhile, there is no ~~organized~~ organised screening.

Formatted: English (United Kingdom), Highlight

Formatted: English (United Kingdom)

Formatted: English (United Kingdom)

Declarations

Ethics and Consent to Participate statement

The Ethical Committee of the Jordi Gol i Gurina Foundation (http://www.idiapjordigol.com/) approved this study.

Consent to Publish Statements

Not applicable.

Competing Interest Statement

The authors declare that they have no conflict of interest.

~~Au~~ Contributions ~~(if there is more than one author)~~

MTB participated in the design of the economic evaluation, carried out the analysis, wrote the first draft of the manuscript, and reviewed the present manuscript. AAP, SSJ, and MDS participated in the design and~~,~~ analysis and collaborated in the preparation and revision of the present manuscript. JMMD, DRC, ARM, JMBS, NSS, and PHV collaborated in the design of this analysis and the revision of the present manuscript.

Availability of Data and Materials Statement

The dataset supporting the conclusions of this article will not be shared because it is not a public dataset. This dataset belongs to ~~three~~ 3 Primary Care Trusts in Catalonia ~~which~~ that are part of the Catalonian Public Health System. However, you ~~could~~ can contact the main investigator of the clinical trial (aacera.mn.ics@gencat.cat) ~~to consult the~~ for access to it.

Formatted: English (United Kingdom)

Formatted: English (United Kingdom)

Abbreviations

ASSIR	Reproductive and Sexual Health Primary Care Unit
BHCA	Basic Health Care Area

HPV	Human Papillomavirus
ICER	Incremental Cost-Effectiveness Ratio
IDIAP	Primary Care Research University Institute
IG1	Intervention group 1 that in~~l~~cludes a ~~personalized~~ personalised invitation letter to participate in the screening signed by the patient's primary care physician and professionals of the corresponding public health ~~center~~centre.
IG2	Intervention group 2 that in~~l~~cludes the same letter of invitation sent in the IG1 as well as an informative leaflet on the prevailing screening of cervical cancer
IG3	Intervention group 3 that in~~l~~cludes the same intervention as the one performed in IG2, complemented by a phone call 3 days prior to the appointment indicated in the letter of invitation as a reminder of the visit
NIG	No intervention group with current opportunistic screening
SAP	Primary Care Service

Formatted: English (United Kingdom)

Formatted: English (United Kingdom)

Funding

This p~~P~~roject was financed by t~~T~~he Spanish Ministry of Health (ISCIII Exp. PI10/01275) in the 2010 grant call of the Strategic Health Action Plan~~,~~ within the framework of the National Plan for Scientific Research, Development, and Innovative Technology. It was co-financed by the European Union~~,~~ through European Funds for Regional Development (FEDER). Partial support for the development of this work was received from Spanish public grants from the Instituto de Salud Carlos III (RTIC RD06/0020/0095, RD12/0036/0056, and CIBERESP) and from the Agència de Gestió d'Ajuts Universitaris i de Recerca (grants AGAUR 2014SGR1077 and 2014SGR2016).

Acknowledgements

The authors acknowledge the useful comments from an external blind reviewer.

References

1. Ferlay J, Soerjomataram I, Ervik M, Dikshit R, Eser S, Mathers C, et al. GLOBOCAN 2012 v1.0, Cancer Incidence and Mortality Worldwide: IARC CancerBase No. 11 [Internet]. Lyon, France: International Agency for Research on Cancer; 2013. [Available from: http://globocan.iarc.fr, accessed on day/month/year].[Accessed on January 2016].
2. Clèries R1, Esteban L, Borràs J, Marcos-Gragera R, Freitas A, Carulla M, et al. Time trends of cancer incidence and mortality in Catalonia during 1993-2007. Clin Transl Oncol 2014;16(1):18-28.
3. Luengo-Fernández R, Leal J, Gray A, Sullivan R. Economic burden of cancer across the European Union: a population-based cost analysis. The Lancet 2013; 14(12):1165-1174.
4. Lew J-B, Howard K, Gertig D, Smith M, Clements M, Nickson C, et al. Expenditure and resource utilisation for cervical screening in Australia. BMC Health Services Research 2012;12:446. DOI: 10.1186/1472-6963-12-446.
5. Anttila A, Ronco G. Description of the national situation of cervical cancer screening in the member states of the European Union. *European journal of cancer (Oxford, England : 1990)* 2009;45:2685–708.
6. European CDC (ECDC): Guidance for the introduction of hpv vaccines in European Union countries. *Stockholm* 2008.
7. Everett T, Bryant A, Griffin MF, Martin-Hirsch PPL, Forbes CA, and Jepson RG. . Interventions targeted at women to encourage the uptake of cervical screening. Cochrane Database Syst Rev. 2011; (5): CD002834.
8. Sabatino SA, Lawrence B, Elder R, Mercer SL, Wilson KM, DeVinney B, et al. Effectiveness of interventions to increase screening for breast, cervical, and colorectal cancers: nine updated systematic reviews for the guide to community preventive services. *American journal of preventive medicine* 2012;43:97–118.

Formatted: Highlight

9. Dinkelspiel H, Fetterman B, Poitras N, Kinney W, Cox JT, Lorey T, et al. Screening history preceding a diagnosis of cervical cancer in women age 65 and older. *Gynecologic oncology* 2012; 126:203–6.
10. Acera A, Manresa JM, Rodriguez D, Rodiguez A, Bonet JM, Trapero-Bertran M, Hidalgo P, de Sanjosé S. Increased cervical cancer screening coverage a successful intervention: the CRIVERVA study. BMC Women's Health, 2016.; under evaluation.

Formatted: Highlight

11. Acera A, Rodriguez A, Trapero-Bertran M, Soteras P, Sanchez N, Bonet JM, et al. Economic evaluation of three populational screening strategies for cervical cancer in the county of Valles Occidental: CRICERVA clinical trial. *BMC health services research* 2011;11:278.
12. Georgalis L, de Sanjosé S, Esnaola M, Bosch FX, Diaz M. Present and future of cervical cancer prevention in Spain: a cost-effectiveness analysis. Eur J Cancer Prev 2015 Sep 15. [Epub ahead of print].
13. Atenció a la Salut Sexual i Reproductiva) Cerdanyola. Productes Intermedis. Any 2014. Area Metropolitana Nord. Institut Català de la Salut. Generalitat de Catalunya
14. Instituto Nacional de Estadística. Actualización de rentas con el IPC general (sistema IPC base 2011) para periodos anuales completos. INE, 2015. <http://www.ine.es/calcula/calcula.do;jsessionid=2397A2370F415D7DB8B70F5C887A2A29.calcula03>.
15. Gray AM, Clarke PM, Wolstenholme JL, Wordsworth S. Applied methods of cost-effectiveness analysis in health care. Oxford University Press, 2011.UK.
16. Hyndman JC, Straton JA, Pritchard DA, Le Sueur H. Cost-effectiveness of interventions to promote cervical screening in general practice. Australian and New Zeland Journal of Public Health 1996;20(3):272-277.
17. Broberg G, Miao Jonasson J, Ellis J, Gyrd-Hansen D, Anjemark B, Glantz A, et al. Increasing participation in cervical cancer screening: Telephone contact with long-term non-attendees in Sweden. Results from RACOMIP, a randomized controlled trial. International Journal of Cancer 2013;133(1):164-171.

18. Heranney D, Fender M, Velten M, Baldauf JJ. A prospective randomized study of two reminding strategies: telephone versus mail in the screening of cervical cancer in women who did not initially respond. Acta Cytologica 2011;55(4):334-340.

19. Kesic V, Poljak M, Rogovskaya S. Cervical cancer burden and prevention activities in Europe. Cancer Epidemiology, Biomarkers & Prevention 2012; 21(9):1423-1433.

20. Anttila A, von Karsa L, Aasmaa A, Fender M, Patnick J, Rebolj M, et al. Cervical cancer screening policies and coverage in Europe. European Journal of Cancer 2009; 45(15):2649-2658.

21. Arbyn M, Anttila A, Jordan J, Ronco G, Schenck U, Segnan N, et al. European guidelines for quality assurance in cervical cancer screening. 2nd edition. 2008. Brussels, Luxemburg: European Community, Office for Official publications of the European Communities.

22. Nicula FA, Anttila A, Neamtiu L, Primic Zakelj M, Tachezy R, Chil A, et al. Challenges in starting organised screening programmes for cervical cancer in the new member states of the European Union. European Journal of Cancer 2009; 45(15):2679-2684.

23. Virtanen A, Anttila A, Luostarinen T, Malila N, Nieminen P. Improving cervical cancer screening attendance in Finland. International Journal of Cancer 2015;136(6):E667-E684.

24. Spadea T, Bellini S, Kunst A, Stirbu I, Costa G. The impact of interventions to improve attendance in female cancer screening among lower socioeconomic groups: A review. Preventive Medicine 2010; 50(4):159-164.

25. De Sanjosé S, Garcia AM. *Virus del papiloma humano y cáncer: epidemiología y prevención*. Monography. Madrid: Sociedad Española de Epidemiologia 2006;1–144.

26. Siebert U, Sroczynski G, Hillemans P, Engel J, Stabenow R, Stegmaier C, et al. The German cervical cancer screening model: development and validation of a decision-analytic model for cervical cancer screening in Germany. European Journal of Public Health 2006;16(2):185-192.

Table Click here to download Table Tables CRICERVA CEA_FIN_050816_MTB.docx

Table 1 Population included in the CRICERVA project

**Population**	**IG1** (letter)	**IG2** (letter + leaflet)	**IG3** (letter + leaflet + phone call)
**Target population**			
<40	3251	2847	3799
40-49	2444	2146	2812
50-59	1784	1900	2406
≥60	1489	1276	2010
TOTAL	8968	8169	11027
**Poorly screened population**[a]			
<40	1113	948	1449
40-49	1224	974	1750
50-59	798	754	1260
≥60	1062	925	1629
TOTAL	4197	3601	6088
**Answer to the intervention**[b]			
<40	879	862	1079
40-49	861	683	1050
50-59	611	589	932
≥60	604	491	963
TOTAL	2955	2625	4024

**Women screened by the intervention**[c]			
<40	449	392	576
40-49	512	381	665
50-59	314	318	584
≥60	303	276	571
TOTAL	1578	1367	2396

[a] Invited to participate because of last screening before of three and a half years ago

[b] Those women who are contacted through any of the interventions and are willing to attend the GP visit

[c] Number of women who finally attend th GP visit

Table 2 Management costs

Interventions	Costs(€ 2014)
NIG	Includes one visit with 15 minutes of a nurse/midwife (35.64€), one citology (21.78€)and one HPV test (28.71€) (Total: 86.13€)
IG1	Cost of the no intervention plus a letter and its posting (0.16€) and the office time (0.33€) (Total: 86.62€)
IG2	Cost of the no intervention plus a letter and its posting (0.16€) plus a leaflet and its posting (1€),and the officer time (0.33€+0.35€) (Total: 87.97€)
IG3	Cost of the no intervention plus a letter and its posting (0.16€) plus a leaflet and its posting (1€),a reminding call (0.30€) and the officer time (0.33€+0.35€+0.83€) (Total: 89.10€)

Source: References 12 and 13

Table 3 Sociodemographic and behavioural characteristics of women interviewed

Characteristic	No Intervention Group (NIG)	Intervention groups			TOTAL	P
		Letter (IG1)	Letter + leaflet (IG2)	Letter + leaflet + phone call (IG3)		
**Interviewed**	857	807	848	1011	3523	
**Age, mean (SD)**	50.8 (12.7)	49.5 (12.1)	50.0 (12.4)	51.1 (12.0)	50.4 (12.3)	**0.018**
**Spanish nationality**	827 (96.5%)	744 (92.2%)	768 (90.7%)	900 (89.1%)	3239 (92.0)	<0.001
**Educational level**						
None	43 (5.1%)	82 (11.9%)	64 (9.5%)	71 (8.0%)	260 (8.4%)	<0.001
Primary	504 (30.1%)	380 (27.5%)	377(28.0%)	423 (23.8%)	1684 (27.2%)	
High School/ University	291(17.3%)	229(16.5%)	231(17.2%)	395(22.2%)	1146(18.5%)	
**Marital status-married**	594 (70.3%)	518 (74.6%)	513 (76.5%)	666 (74.7)	2291 (73.9%)	0.037
**Number of children**						
0	93 (11.0%)	97 (14.0%)	74 (11.0%)	114 (12.8%)	379 (12.2%)	0.002
1-2	443 (52.5%)	404 (58.4%)	394 (58.5%)	524 (58.9%)	1765 (57.0%)	
>2	308 (36.5%)	191 (27.6%)	205 (30.5%)	251 (28.2%)	955 (30.8%)	
**Lag time since last Pap screening**						
1-3 years	417 (48.7%)	348 (43.8%)	369 (44.6%)	421 (42.1%)	1555 (44.7%)	0.002
4-6 years	322 (37.6%)	282 (35.5%)	294 (35.6%)	391 (39.1%)	1289 (37.1%)	
never	117 (13.7%)	164 (20.7%)	164 (19.8%)	189 (18.9%)	634 (18.2%)	
**Reasons for non-attendance to screening for women with no previous Pap**						
Fear and dislike	23 (19.8%)	65 (41.1%)	68 (42.2%)	73 (40.3%)	229 (37.2%)	<0.001
Uninformed	91 (78.4%)	84 (53.2%)	80 (49.7%)	98 (54.1%)	353 (57.3%)	
Other	2 (1.7%)	9 (5.7%)	13 (8.1%)	10 (5.5%)	34 (5.5%)	

The questionnaires completed for the intervention groups were carried out during routine medical visits. For the non-intervention group the questionnaires were completed at the end of the study by appropriately trained personnel during a telephone call.

Source: Acera et al (forthcoming 2016)

Table 4 Cost-effectiveness analysis results over the CRICERVA study for all ages

Group	Cost	Incremental coverage (%)	ICER(1)	ICER(2)
No intervention (NIG)	86.13€			
IG1 (letter)	86.62€	17.6%	2.78	2.78 (IG1 vs NIG)
IG2 (letter + leaflet)	87.97€	16.7%	11.02	Dominated (IG2 vs IG1)
IG3 (letter + leaflet + phone call)	89.11€	21.7%	13.73	60.73 (IG3 vs IG1)

(1) Incremental cost-effectiveness ratio of each intervention group compared with the no intervention (opportunistic screening) group expressed as € per 1% coverage.

(2) Incremental cost-effectiveness ratio of one intervention compared with the next least expensive strategy expressed as € per 1% coverage

Table 5 Cost-effectiveness analysis results over the CRICERVA study by age group

**Age**	**Incremental coverage (%)**	**ICER(1)**	**ICER(2)**
**Women < 40**			
No intervention (NIG)			
IG1 (letter)	13.8%	3.55	3.55 (IG1 vs NIG)
IG2 (letter + leaflet)	13.8%	13.33	more expensive (IG2 vs IG1)
IG3 (letter + leaflet + phone call)	15.2%	19.60	177.86 (IG3 vs IG1)
**Women 40-49**			
No intervention (NIG)			
IG1 (letter)	20.9%	2.34	2.34 (IG1 vs NIG)
IG2 (letter + leaflet)	17.8%	10.34	Dominated (IG2 vs IG1)
IG3 (letter + leaflet + phone call)	23.6%	12.63	92.22 (IG3 vs IG1)
**Women 50-59**			
No intervention (NIG)			
IG1 (letter)	17.6%	2.78	2.78 (IG1 vs NIG)
IG2 (letter + leaflet)	16.7%	11.02	Dominated (IG2 vs IG1)
IG3 (letter + leaflet + phone call)	24.3%	12.26	37.16 (IG3 vs IG1)
**Women ≥ 60**			
No intervention (NIG)			
IG1 (letter)	20.3%	2.41	2.41 (IG1 vs NIG)
IG2 (letter + leaflet)	21.6%	8.52	103.85 (IG2 vs IG1)
IG3 (letter + leaflet + phone call)	28.4%	10.49	16.76 (IG3 vs IG1)

(1) Incremental cost-effectiveness ratio of each intervention group compared with the no intervention (opportunistic screening) group expressed as € per 1% coverage.

(2) Incremental cost-effectiveness ratio of one intervention compared with the next least expensive strategy expressed as € per 1% coverage

Elsevier english editing certificate

Click here to access/download
Supplementary Material
Elsevier_English editing certificate.pdf
